# Dynamic Modulation of Thymic MicroRNAs in Response to Stress

**DOI:** 10.1371/journal.pone.0027580

**Published:** 2011-11-16

**Authors:** Serkan Belkaya, Robert L. Silge, Ashley R. Hoover, Jennifer J. Medeiros, Jennifer L. Eitson, Amy M. Becker, M. Teresa de la Morena, Rhonda S. Bassel-Duby, Nicolai S. C. van Oers

**Affiliations:** 1 The Department of Immunology, The University of Texas Southwestern Medical Center, Dallas, Texas, United States of America; 2 The Department of Pediatrics, The University of Texas Southwestern Medical Center, Dallas, Texas, United States of America; 3 The Department of Molecular Biology, The University of Texas Southwestern Medical Center, Dallas, Texas, United States of America; 4 The Department of Microbiology, The University of Texas Southwestern Medical Center, Dallas, Texas, United States of America; MRC National Institute for Medical Research, United Kingdom

## Abstract

Physiological stress evokes rapid changes in both the innate and adaptive immune response. Immature αβ T cells developing in the thymus are particularly sensitive to stress, with infections and/or exposure to lipopolysaccharide or glucocorticoids eliciting a rapid apoptotic program. MicroRNAs are a class of small, non-coding RNAs that regulate global gene expression by targeting diverse mRNAs for degradation. We hypothesized that a subset of thymically encoded microRNAs would be stress responsive and modulate thymopoiesis. We performed microRNA profiling of thymic microRNAs isolated from control or stressed thymic tissue obtained from mice. We identified 18 microRNAs that are dysregulated >1.5-fold in response to lipopolysaccharide or the synthetic corticosteroid dexamethasone. These included the miR-17-90 cluster, which have anti-apoptotic functions, and the miR-181 family, which contribute to T cell tolerance. The stress-induced changes in the thymic microRNAs are dynamically and distinctly regulated in the CD4^−^CD8^−^, CD4^+^CD8^+^, CD4^+^CD8^−^, and CD4^−^CD8^+^ thymocyte subsets. Several of the differentially regulated murine thymic miRs are also stress responsive in the heart, kidney, liver, brain, and/or spleen. The most dramatic thymic microRNA down modulated is miR-181d, exhibiting a 15-fold reduction following stress. This miR has both similar and distinct gene targets as miR-181a, another member of miR-181 family. Many of the differentially regulated microRNAs have known functions in thymopoiesis, indicating that their dysregulation will alter T cell repertoire selection and the formation of naïve T cells. This data has implications for clinical treatments involving anti-inflammatory steroids, ablation therapies, and provides mechanistic insights into the consequences of infections.

## Introduction

The thymus is the critical organ required for the production of the αβ T lymphocytes of the immune system, a developmental process maintained throughout life [1], [2], [3]. Selection mechanisms within the thymus ensures the development of naïve T cells capable of recognizing foreign peptides bound to self-major histocompatibility complex (MHC) molecules, which are presented in the peripheral lymphoid organs [1]. These self-restricted T cells are essential for effective immune responses to infections. Interestingly, developing thymocytes are exquisitely sensitive to physiological and pathological stress, which causes a rapid programmed cell death termed apoptosis [4], [5]. This response is evident during infections, as well as in humans undergoing immunosuppressive treatments, radiation therapy, and/or surgery [6], [7], [8], [9]. Even milder stress such as pregnancy, emotional anxiety, malnutrition, or alcoholism can reduce thymic cellularity [10], [11], [12], [13]. All these forms of stress increase the systemic and/or intrathymic production of glucocorticoids, which are major regulators of lymphocyte apoptosis [14], [15]. In the case of bacterial infections, the innate immune system responds to pathogen associated molecular patterns (PAMPs), releasing inflammatory cytokines, such as interferon, IL-1β, TNF-α, IL-6, and leukemia inhibitory factor (Lif) [11], [16], [17]. The IL-6 family (oncostatin-M, IL-6, leukemia inhibitory factor) induces the production of corticosteroids (glucocorticoids (GC)) from the hypothalamus, pituitary-adrenal (HPA) axis and intrathymically [18], [19], [20]. Influenza virus also elevates glucocorticoids systemically, albeit via undefined mechanisms [21]. Clinically, synthetic glucocorticoids (e.g. dexamethasone, methylprednisone) are routinely used to suppress immune functions in transplant recipients, during autoimmune and systemic inflammatory disorders, and for treating certain malignancies [22], [23], [24]. The effects of these treatments on thymopoiesis are becoming better understood with the identification of down-regulated genes that are coupled to T cell development, cell cycle, and cell activation [25]. Functionally, GCs, lipophilic steroids, diffuse across the plasma membrane and complex a specific member of the nuclear receptor family, designated nuclear receptor 3 group 1 (NR3C1) [4]. This glucocorticoid-bound receptor complex dimerizes and translocates into the nucleus where it interacts with glucocorticoid response elements (GREs), culminating in the trans-activation of apoptotic effector genes [4], [14], [26], [27]. Thymocytes are very sensitive to glucocorticoid-induced cell death because of high levels of NR3C1 [4], [28].

MicroRNAs (miRs) are key, stress-responsive regulators in the immune, hematopoietic, cardiac, thyroid, and auditory systems [29], [30], [31], [32], [33]. They are small, non-coding RNA molecules (20–25 nucleotides in length) that repress global mRNA translation and/or induce mRNA degradation (reviewed in [33], [34]). Within hours of exposure to GCs, the protein levels of three miR-processing enzymes, Drosha, Pasha/DiGeorge Critical Region 8 (DGCR8), and Dicer are reduced significantly in thymocytes [35]. These enzymes/miR-binding proteins generate small miRs from the larger pre-miRs processed from various RNA transcripts. The targeted deletion of Dicer and Drosha in developing thymocytes causes a significant loss of cells, and results in a lethal lymphoid inflammatory disorder, partly from the loss of T regulatory cells [36], [37], [38]. Thus, a transient loss of Dicer and Drosha following GC treatment could modulate T cell development. There are several additional miRs with key roles in thymopoiesis. MiR-181a is highly expressed in DP thymocytes, representing 15% of the total miR pool [39], [40], [41]. This particular miR targets genes that regulate T cell receptor signaling pathways [40], [41]. The normally high levels of miR-181a maintain T cell tolerance to self-peptide/MHC molecules, with a reduction in this miR increasing the number of self-reactive T cells [42]. Less is known about the role of the three other miR-181 family members (miR-181b, c, and d), two of which are expressed in developing thymocytes [39]. The targeted deletion of miR-146a in T regulatory cells results in rampant autoimmunity [43]. While little is known about the effects of stress on the thymic miRs, certain miRs are up regulated in innate effector cells following bacterial infections and/or LPS exposure (MiR-146a, MiR-155) [29], [43], [44], [45], [46], [47]. Given the critical function of T cells in the immune system, the modulation of miRs during thymopoiesis could have important clinical consequences.

Herein, we describe 18 thymic miRs that are dysregulated >1.5 fold in mice exposed to LPS or dexamethasone for one to three days. Many of these miRs exhibited a distinct responsiveness in immature versus mature thymocyte subsets, with the CD4^−^CD8^+^ subset the most refractory to miR down-regulation. MiR-181d, a member of the miR-181 family, had a 5–15 fold reduced expression following LPS or dexamethasone treatment, suggesting an important functional role for this miR in thymopoiesis. By performing luciferase reporter assays with defined targets, miR-181d targeted a similar set of genes as miR-181a. In addition, miR-181d uniquely targeted Lif. These experiments indicate that stress can have a profound impact on thymopoiesis, altering the expression of miRs involved in apoptosis, tolerance, and proliferation.

## Results

### LPS and synthetic steroids reduce CD4^+^CD8^+^ thymocyte numbers

**Figure 1:**
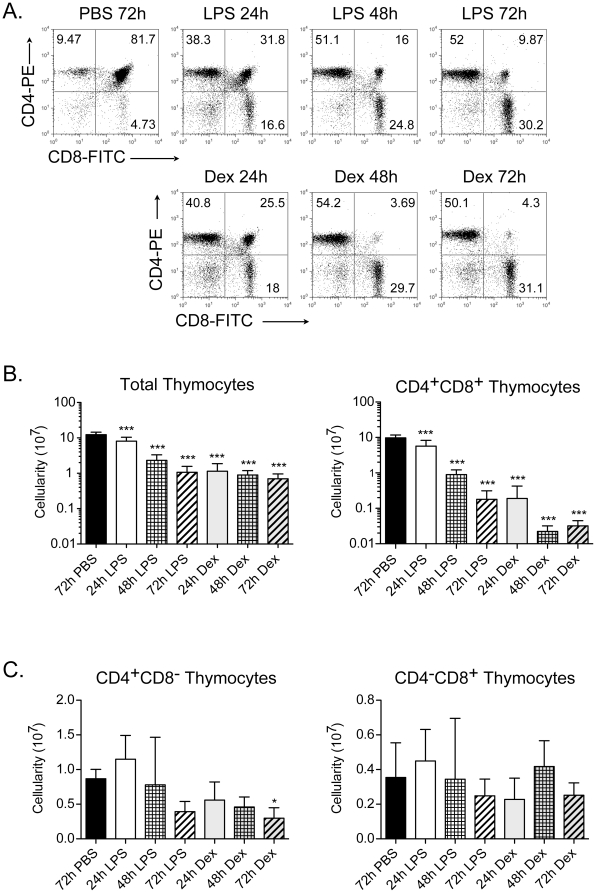
Lipopolysaccharide and dexamethasone deplete immature CD4^+^CD8^+^ thymocytes. A) Thymocytes were isolated from either control (PBS injected, t = 72 h), or LPS and dexamethasone (Dex) injected mice (t = 24, 48 and 72 h). The cells were stained with fluorochrome-labeled anti-CD4 and anti-CD8 mAbs and analyzed by flow cytometry. The percentage of CD4^−^CD8^−^ (DN), CD4^+^CD8^+^ (DP), CD4^+^CD8^−^ (CD4 SP), and CD4^−^CD8^+^ (CD8 SP) subsets are provided in each quadrant of the dot plot profiles. B) The total thymic cellularity and the number of CD4^+^CD8^+^ cells were calculated at 24, 48, and 72 h post-injection and are shown in a log scale. C) The absolute number of mature CD4^+^CD8^−^ and CD4^−^CD8^+^ SP cells after PBS, LPS, and Dex treatments were calculated at t = 24, 48 and 72 h post-injection. Data are representative of mean +/− SD from at least 8 mice per group (*p<0.05, **p<0.01, *** p<0.001, versus PBS control; one-way ANOVA analyses).

Stress, resulting from infections, induces thymocyte apoptosis and suppresses peripheral T cell responses [16], [17], [18], [19], [27]. A single intraperitoneal (IP) injection of 100 µg lipopolysaccharide (LPS), used to mimic a bacterial infection, caused a significant reduction in the percentage of CD4^+^CD8^+^ (DP) thymocytes within 24 h (Figure 1A). These DP cells were almost completely eliminated by 72 h (Figure 1*A*). The reduced percentage of DP cells results from LPS-induced elevations in glucocorticoids [19]. Consistent with this, an IP injection of 60 µg dexamethasone (Dex), a synthetic glucocorticoid, also caused a significant loss of the DP thymocytes that was revealed within 24 h (Figure 1A). While both treatments increased the percentage of CD4^+^CD8^−^ and CD4^−^CD8^+^ (SP) thymocytes, the absolute numbers of these SP cells were not altered (Figures 1A–C). This indicated that the DP subset was selectively targeted (Figures 1B–C). Unlike thymocytes, the consequences of LPS and Dex injections on peripheral lymphocytes were distinct. LPS increased the number of mature B cells (B220^+^) at 48 h while Dex caused a depletion of both B and T cells at specific time points (Figure S1A–D). Taken together, these results indicate that DP thymocytes are acutely sensitive to stress induced by both LPS and Dex.

### Selected thymic microRNAs are differentially regulated in diverse tissues following stress

**Figure 2:**
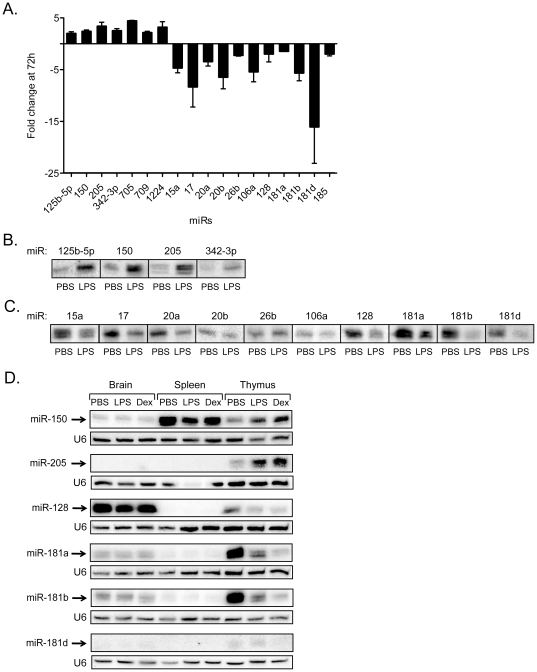
Stress responsive signature microRNAs in thymic tissue. A) Graph shows 18 distinct miRs with a statistically significant fold change (p<0.05) in expression. Fold change indicates the change in miR expression in LPS-treated sample relative to PBS-treated sample. B) Northern blots of 4 miRs that were up regulated in thymic tissue 72 h after LPS injections. C) Northern blots were performed on 10 miRs that were down-regulated 72 h after LPS exposure. D) Differential expression of stress responsive miRs in diverse tissues. Male mice were injected with PBS, LPS or Dex. Total RNA was isolated from the brain, spleen, and thymus of control (PBS) and LPS- or Dex- treated mice at 72 h post-injection. The individual miRs (miR-150, miR-205, miR-128, miR-181a, miR-181b, miR-181d) were detected by Northern blotting. The relative amounts of a control RNA were determined by blotting for U6.

Three miR processing enzymes, Drosha, Pasha/DGCR8, and Dicer, are significantly reduced in thymocytes within 6–12 h of glucocorticoid treatment, leading to an overall reduction in the miRs in the thymus [35]. Our findings suggested that the most dramatic changes in thymocyte subset composition occurred at 72 h. To profile the miRs at this time point, RNA was prepared from the thymus of mice 72 h after treatment with PBS or LPS. Murine microRNA arrays were used to profile over 600 miRs (Data Set S1). Seven and 11 distinct miRs were up- and down-regulated, respectively, at levels greater than 1.5-fold (p<0.05, 3 mice/group, 6 mice total) (Figure 2A, Figure S1E). The changes in 14 of the 18 miRs were confirmed by northern blotting using probes specific for each miR, with U6 RNA as an internal control for RNA levels. MiR-125b-5p, miR-150, miR-205, and miR-342-3p were consistently up regulated in the thymic tissue from the LPS injected mice (Figure 2B). MiR-15a, miR-17, mir-20a, miR-20b, miR-106a, miR-128, miR-181a, miR-181b, and miR-181d were consistently down regulated (Figure 2C). While miR-26b appeared down regulated, based on the microRNA array data, northern blots did not consistently reveal this change (Figure 2C). MiR-1224, miR-709, and miR-705 could not be characterized by northern blotting due to high, non-specific background issues with the probes (data not shown). MiR-185 gave a weak signal when assessed by northern blotting (data not shown). Of note, the fold changes revealed in the miR microarrays were much greater than that identified by northern blotting because the arrays are more sensitive, specific, and quantitative [48].

We next examined whether any of the miRs identified in the thymus were stress responsive in other tissues [39]. Northern blotting was performed using RNA isolated from the heart, kidney, liver, brain, spleen, and thymus of PBS-, LPS- and Dex-treated mice at 72 h post-injection (Figure 2D, Figure S2B). While miR-150 was identified in both the spleen and thymus, it only appeared up regulated in the thymic tissue following LPS or Dex treatments (Figure 2D). MiR-205 and miR-181d were uniquely expressed and stress responsive in the thymic tissue. MiR-128, identified in the brain and thymus, was only down regulated in the latter following a stress response. Of the miR-181 family members that were stress responsive in the thymus, miR-181a and miR-181b were also weakly expressed in the brain and spleen. Many of the other miRs were stress responsive in diverse tissues. For example, miR-15a, miR-17, miR-20b, and miR-26b were stress responsive in nearly every tissue examined (Figure S2C). MiR-342-3p, primarily brain- and spleen- specific, was reduced upon LPS and Dex treatment. MiR-20b, miR-106a and miR-150 were stress responsive, but were only identified in the thymus and spleen. MiR-125b-5p was highly expressed in the brain and heart, but it was detected at low levels in other tissues. Taken together, our experiments reveal that many of the miRs identified in the thymus are stress responsive in different tissues.

**Figure 3:**
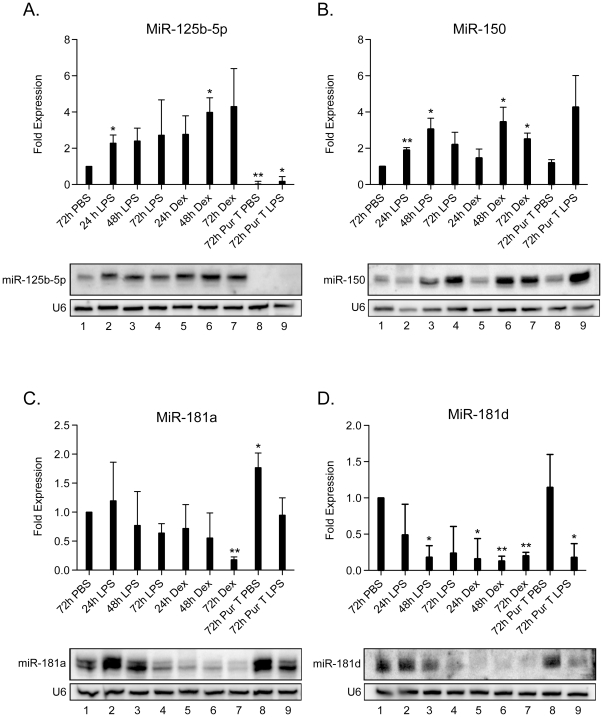
Stress responsive changes in thymic miR profiles are time dependent. Total RNA was isolated from thymic tissue prepared from PBS- (lane 1), LPS- (lanes 2–4), and Dex- (lanes 5–7) treated mice at 24 h (lanes 2, 5), 48 h (lanes 3, 6), and 72 h (lanes 1, 4, 7). In lanes 8–9, RNA was prepared from purified T cells isolated from the thymus. Northern blots were performed for the selected miRs A) Mir-125, B) MiR-150, C) MiR-181a, and D) MiR-181d. The samples were quantified by phoshorimager analyses, following background subtraction, and normalizing for total RNA amounts with a U6 probe. Data shown are mean +/− SD of relative fold changes in miR expression levels of PBS- versus LPS- or Dex-treated samples from 3 to 5 independent Northern blots (*p<0.05, **p<0.01, *** p<0.001, versus PBS control; unpaired Student t-test).

The miR array profiling was undertaken at a single time point (72 h). To determine the temporal alterations in miR levels following LPS or Dex treatments, northern blots were performed with RNA extracted from the thymus at 24, 48, and 72 h. MiR-125b-5p and miR-150 exhibited a transient, 2-fold increase 24 h post-LPS and -Dex injections (Figure 3A, lanes 2 and 5 versus 1). MiR-150 continued to increase and plateaued between 48 and 72 h (Figure 3A, lanes 6–7). Unlike miR-150, miR-125b-5p was primarily expressed in stromal tissue, as it was not detected in purified thymocytes (lanes 8–9). This is consistent with our microarray data comparing differential miR expression in the thymus between wild type and CD3ε^−/−^ mice (Figure S2A). MiR-181a transiently increased in the LPS-treated mice at 24 h, but thereafter dropped 1- and 5-fold upon LPS and Dex treatments, respectively (Figure 3C, lanes 4–7). MiR-181d exhibited the most dramatic down-regulation after both LPS and Dex treatments; with a 5–15 fold reduction by 72 h (Figure 3D, lanes 3, 5–7). The other miRs exhibited time dependent changes in expression (Figure S3). In summary, at least 18 distinct miRs expressed in the thymus are dynamically regulated following LPS and Dex induced stress responses.

### MicroRNA expression patterns are differentially affected by stress in specific thymocyte subsets

**Figure 4:**
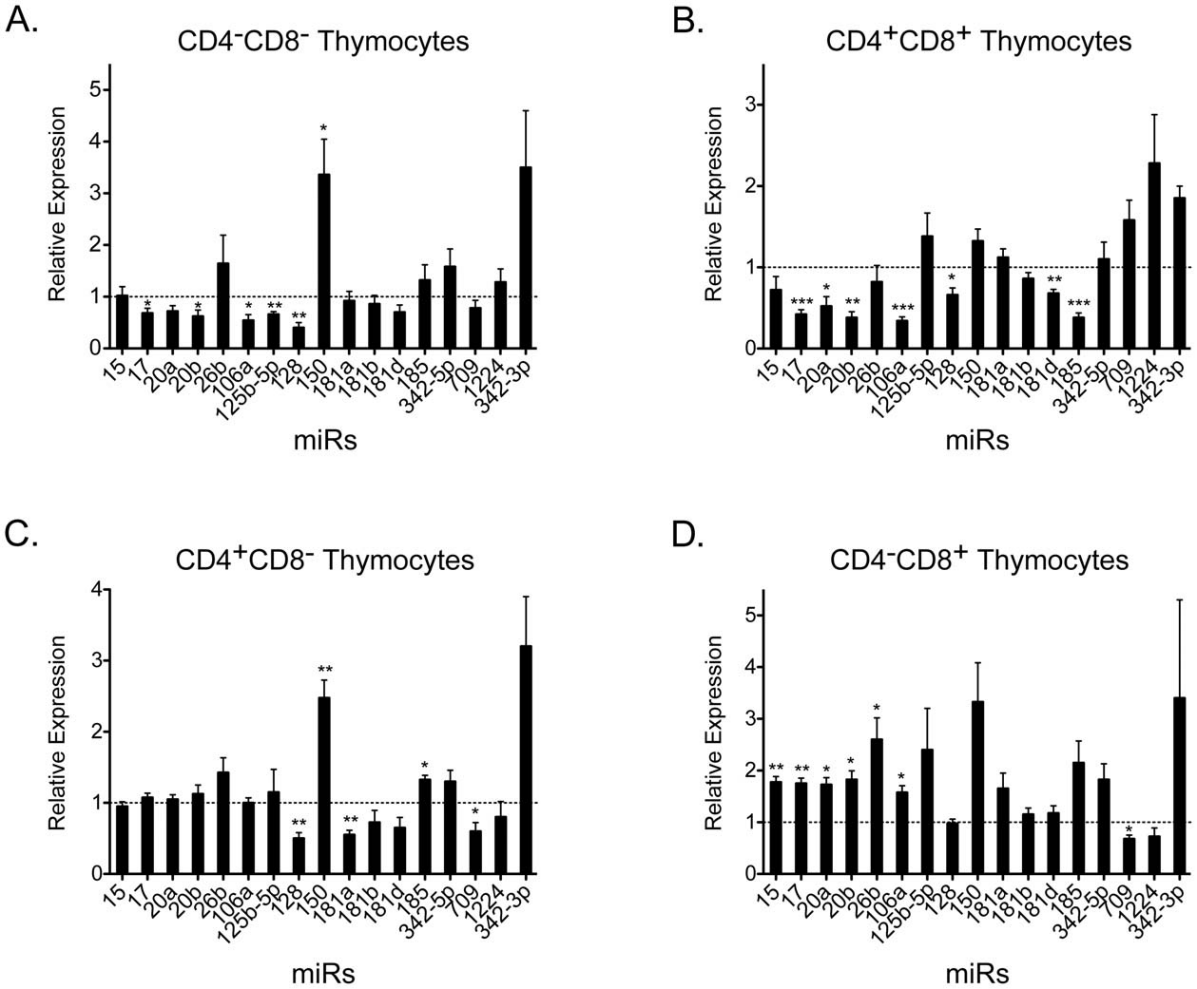
Stress responsive thymic miRs are differentially regulated in thymocyte subsets. Thymocytes from PBS and LPS injected mice (72 h) were sorted into A) CD4^−^CD8^−^ (DN), B) CD4^+^CD8^+^ (DP), C) CD4^+^CD8^−^ (CD4 SP), and D) CD4^−^CD8^+^ (CD8 SP) subsets using high speed cell sorting. RNA was isolated from the sorted subpopulations. Real-time microRNA RT-PCR was used to detect the levels of the indicated miRs in various subsets. The vertical line on the figure indicates the control (PBS) threshold set at 1. Data represent the averaged fold changes in miR expression levels of PBS- versus LPS-treated samples calculated by ΔΔC_T_ normalized to the endogenous U6 levels, of 4 independent experiments performed in triplicate (*p<0.05, **p<0.01, *** p<0.001, versus the threshold; one sample Student t-test).

To determine whether the stress-responsive miRs were equivalently or differentially regulated in particular T cell subsets, thymocytes from control and LPS-injected mice were sorted into the DN, DP, CD4 SP, and CD8 SP subsets. MiR profiling was performed using quantitative real-time PCR (RT-PCR) with miR-specific probes (Figure 4A–D). With the exception of miR-150 and miR-342-3p, the majority of the miRs were down regulated in the DN subset. The reduced expression of the miR-17-90 cluster and the miR-181 family was even more pronounced in the DP population (Figure 4B). Comparing all the thymocyte subsets, the CD8 SP was the most divergent, with the miR-17-90 cluster up regulated 2-fold and the miR-181 group unaffected by the LPS treatment. It should be noted that the down-regulation of the miR-181 family was not as obvious with the RT-PCR assays as with the arrays. This is because miR-181a, miR-181b, miR-181c, and miR-181d are very conserved, with only 1–4 bp differences within this family. Since the PCR amplification is not as stringent as the arrays, and it is likely that miR-181a can be amplified to a small degree when using a miR-181d specific primers, causing a less obvious down-regulation. In summary, LPS exposure induces a dynamic, time-dependent modulation of miRs that is distinct in each of the thymocyte subsets.

### Mir-181a and miR-181d have both overlapping and distinct gene targets

**Figure 5:**
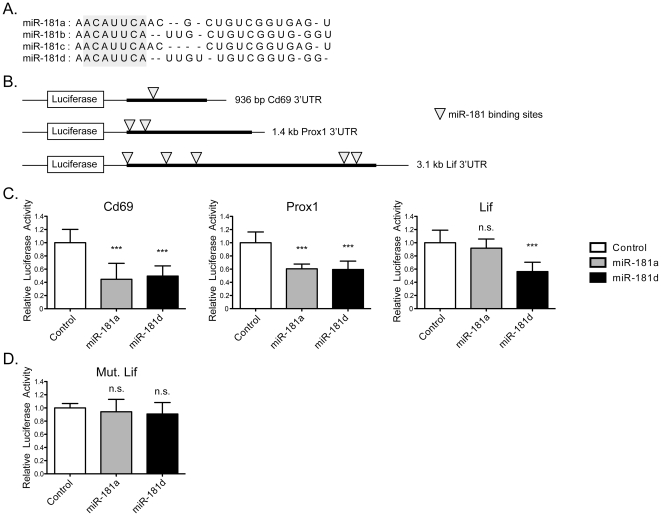
Target specificity of miR-181 family members. A) Homology of miR-181 family members with the seed sequences shaded. B) Schematic representation of the reporter constructs and predicted binding sites of miR-181 family in the 3′ untranslated region of Cd69, prox1, and Lif. C–D) Luciferase reporter assays. A beta-galactosidase expressing vector, and luciferase reporter constructs containing the 3′ untranslated region of the murine Cd69, Prox1, and Lif (WT or with point mutations in two putative miR-181 binding sites (Mut. Lif) genes were co-transfected along with vector alone or vectors expressing mir-181a or miR-181d. Firefly luciferase activity was normalized to beta-galactosidase activity. Each graph represents mean +/− SD of the ratio of the normalized luciferase activity in miR-181 and control vector transfections from three independent experiments, with each sample tested in triplicate (n.s. = not significant, *p<0.05, **p<0.01, *** p<0.001, versus vector alone, unpaired Student t-test).

The stress-responsive thymic miRs identified in our profiling were grouped into subsets based on their collective functions and/or sequence similarity. Many genes, linked to specific cellular programs such as stress responses or cell cycle, are down regulated in the thymus following LPS exposure [25]. We used public miR target prediction databases, such as TargetScan [49], Miranda [50], PicTar [51], and RNAhybrid [52], to identify potential stress-responsive genes targeted by the miRs identified (Table 1). The miR-181 family members have overlapping targets such as Cd69, Prox1, Bcl-2, dual specificity phosphatases, and protein tyrosine phosphatases (Table 1). MiR-181a is required for effective T cell tolerance to self-antigens. However, little is known about its family member, miR-181d. While all murine miR-181 family members have the same seed region (nucleotides 2–8 at the 5′ end), miR-181d is the most divergent based on total pre-miR sequence, and its precise function is unknown (Figure 5A) [53], [54]. MiR-181d was the most dramatically down-regulated miR in our profiling study. Leukemia inhibitory factor (Lif) is a key, stress-responsive gene identified in the thymus [18], [19]. It is a member of the IL-6 cytokine family, expressed by thymic epithelial cells and T lymphocytes, which elevates GC levels following LPS exposure [18], [19], [55]. Interestingly, the 3′ untranslated region (UTR) of Lif contains 5 putative miR-181 binding sites (Figure S4A). To determine whether miR-181a and miR-181d have overlapping or distinct target specificities, the 3′ UTRs of murine Cd69, Prox1, and Lif were cloned downstream of a luciferase reporter construct (Figure 5B). Cd69 and Prox-1 are previously reported miR-181a targets [41], [54], and are used in this study as positive controls for miR-181a. Luciferase reporter assays were performed in the absence or presence of miR-181a or miR-181d, using beta-galactosidase to normalize the transfection efficiency. We found that luciferase activities of both Cd69 and Prox1 were repressed in cells expressing miR-181a or miR-181d, compared to the vector controls (Figure 5C). These findings were consistent with a dose response analysis (Figure S5). However, the over-expression of miR-181d, but not miR-181a, reduced the luciferase activity when Lif was located at the 3′end of the reporter (Figure 5C). This was confirmed by using a mutant version of Lif, which demonstrated no targeting by miR-181d (Fig. 5C). These experiments indicate that miR-181 family members can have both overlapping and distinct gene targets.

**Table 1:** List of putative and experimentally validated targets of stress-responsive thymic miRs.

		Putative Targets		
miR	Fold	Cell Division	Response to Stress	Validated Targets
miR-125b-5p	1.97	Nuf2		Smo, Map2k, Tnf
miR-150	2.38	**Nek6**	**Cxcl1**	c-Myb, Notch4
miR-205	3.36	Birc5, Cdc27		Boris, Pten
miR-342-3p	2.51	Ccnf, Cdc27, Kif11	**Cfb**, **Cxcl9**	Cadm1, Ccnd1, Wnt3a
miR-705	4.41	Ccnf	**Ccl5**, **Cxcl9**	Rbl2, Mapk14, Stat3
miR-709	2.14	Cdc27, Cdc7, Mcm5		Stat3, Zbtb7a, Mapk14
miR-1224	3.17	Birc5, Cdc27, Cdc2a		
miR-15a	−4.66	Cdc27, Cdca7, Mcm5, Ncapg2	**Ccl5**, **Cxcl10**, **Cxcl9**, **Rsad2**,	Bcl-2, Wnt3a, Ccnd1
miR-17	−8.31	Smc2	**Cfb**, **F3**, **Orm1**	Bim, Pten, Mapk14, Stat3
miR-20a	−3.42	Ccne2, Cdca7, Hells, Mcm5, Smc2, **Nek6**	Esco2, **F3**, **Orm1**, **Serpina3n**, **Lif**	Aml1, Mapk14, Zbtb7a
miR-20b	−6.43	Ccne2, Cdca7, Hells, Mcm5, Smc2, **Nek6**	**F3**, **Orm1**, **Serpina3n**, **Lif**	Hipk3, Mylip, Hif1a
miR-26b	−2.21	Birc5, Ccne2, Cdc7, Hells, Kntc1, **Nek6**, Smc2, **Zbtb16**	**Ccl3**, **Cfb**, **Il-6**, **Lif**	Lef1
miR-106a	−5.4	Cdc2a, Mcm5, Smc2, **Nek6**	**Ccl2**, **F3**, **Orm1**, **Rsad2**,	Stat3, Hipk3, Mylip, Mapk14
miR-128	−1.95	**Nek6**	**Ccl2**, **Cxcl13**, **Il-6**, **Rsad2**	Bmi1, Adora2b, E2f3, Tgfbr1
miR-181a	−1.43	Aurkb, Ccnf, Cdc7, Kntc1, Smc2	**Il-6**, **Lif**	Cd69, Tcra, Bcl2, Hoxa11, Ptpn22
miR-181b	−5.64	Aurkb, Ccnf, Cdc7, Kntc1, Smc2	**Il-6**, **Lif**	Aicda, Timp3, Bcl2
miR-181d	−16.04	Aurkb, Ccnf, Cdc7, Kntc1, Smc2	**Il-6**, **Lif**	Nlk, Bcl2
miR-185	−1.95	Cdc27, Mcm5, Ncapg2, **Zbtb16**	**Cxcl10**, **Cxcl5**, **Serpina3n**, **Thbs1**, **Lif**	Cdc42, RhoA, Dnmt1, Cdk6

The experimentally validated targets (from miRTarBase) were compiled for each miR. Putative target genes down- and up-regulated in the top two pathways upon endotoxin-induced thymic atrophy are shown in normal and bold text, respectively.

## Discussion

MiRs have critical roles in the development, differentiation, and expansion of cells in many organ systems. Stress directly affects the biogenesis and functionality of miRs (reviewed in [56]). We describe a set of miRs expressed in the thymus that are differentially regulated following the introduction of systemic stress *in vivo*. The stress was induced by LPS to mimic bacterial infections, and by Dex, the known modulator of LPS-induced thymocyte apoptosis. Many of the miRs present in the thymus that were down regulated following LPS or Dex injection belong to the miR-17-92 cluster, which are anti-apoptotic [57]. The miR-17-92 cluster includes miR-17, miR-20a, miR-20b, and miR-106a. Their down-regulation is consistent with the increased apoptotic program initiated in the thymocytes. Previous work has shown that this same cluster is significantly down regulated in thymocytes within 6 h of glucocorticoid treatment [35]. The short-term 6 h Dex treatments cause a reduction in most miRs due to diminished levels of three key MiR processing enzymes, Dicer, Drosha, and Pasha/DGCR8 [35]. Consequently, no thymic miRs are increased in expression. We examined the longer-term consequences of stress on thymic miRs. As the thymocyte subpopulations are dramatically altered following stress, we also examined the miRs changes in individual thymocyte subsets. For the DP population, the miR-17-92 cluster is reduced at all time points examined. This reduction is unique to the DP subset since the cluster is unchanged in the CD4 SP cells, and up regulated in the CD8 SP population. The loss of this cluster in the DP subset is consistent with the increased programmed cell death evident in these cells. We hypothesize that such a change is necessary to prevent tolerogenic signals in the DP cells during infections when foreign antigens are present. Furthermore, we speculate that the up-regulation of the miR-17-92 cluster in the CD8 SP cells may be important in preventing these cells from dying during infections, providing for a new source of naïve T cells required for maintaining an ongoing immune response. It is interesting to note that even among DP thymocytes, several miRs are up regulated after 24 h of stress. MiR-709, miR-1224, and miR-342-3p/5p are increased several-fold in the DP subset, while miR-150 and miR-342-3p are increased in all the thymocyte subsets. Contrasting these findings, miR-150 is up regulated 2–3.5 fold in the mature SP subsets, while remaining unchanged in the DP population. It is noteworthy that elevations of miR-150 in the hematopoietic system, via retroviral transduction, cause early developmental arrest of B cells and contribute to myeloid leukemia's [58]. This suggests that long-term stress could promote a developmental arrest of the DN cells via the up-regulation of miR-150, the target of which is c-Myb [59]. A number of stress responsive miRs have been characterized in innate effector cells following LPS exposure or infections, including miR-146, and miR-155 [29], [47]. Interestingly, these miRs are not modulated significantly in the thymocyte subsets, implying that stress has distinct effects on the innate versus adaptive immune system. It was noted that the magnitude of miR modulation was not as accurate when using real-time RT-PCR assays compared to the microRNA arrays. This is consistent with the known RT-PCR disadvantages for quantitating miRs [48].

Of the stress responsive miRs identified in the thymus, miR-181d is the most dramatically down-modulated miR. This miR is a member of the miR-181 family, with all four members sharing an almost identical seed sequence. MiR-181d is the most divergent of the four, and is located approximately 100 bp downstream of miR-181c. While both miR-181c and miR-181d are expected to arise from the same polycistronic message, miR-181c is not highly expressed in thymocytes, suggesting additional post-transcriptional processes [39]. In addition, miR-181d and miR-181c are encoded on a chromosome (murine chromosome 8) distinct from the miR-181a and miR-181b cluster, which have undergone gene duplication on two separate chromosomes (murine chromosomes 1 and 2). This difference could also explain why miR-181d expression levels are very susceptible to stress compared to miR-181a or miR-181b. The entire family has a complex regulation and function. Thus, the pre-miR loop nucleotides of miR-181a versus miR-181c result in the differential ability of miR-181a to regulate thymocyte development compared to miR-181c [53]. The stem-loop structure of miR-181d is the most distinct of all members. These findings suggest important functional distinctions between miR-181a and miR-181d. MiR-181d is more highly expressed in induced T regulatory cell populations and pre-B cells, compared to the other family members [39]. Our luciferase reporter assays support distinct functions for this family as Cd69 and Prox1 are targeted by both miR-181a and miR-181d, while miR-181d preferentially targets Lif. This finding is intriguing since the down regulation of miR-181d would increase Lif levels in the thymus. LIF, OSM, and IL-6 induce GC production in LPS-treated mice [18], [19]. Thus, the entire miR-181 family could modulate stress responses by targeting Lif and IL-6, especially since IL-6 is a putative target of these miRs (Table 1). The differentially regulated miRs expressed in the thymus may have functions systemically, especially since miRs are very stable and are detected in the plasma and tissue fluids, indicating that miRs are not restricted to their site of production [60]. From a physiological perspective, we suggest that stress-responsive miRs have crucial roles in the specific elimination of DP thymocytes following stress, thereby preventing tolerance to preventing tolerance to foreign, pathogen-derived antigens.

## Materials and Methods

### Ethics Statement

Procedures involving mice were carried out in accordance with the Institutional Animal Care and Use Committee at the University of Texas Southwestern Medical Center. All animal use adheres to applicable requirements such as the Animal Welfare Act, the Guide for the Care and Use of Laboratory Animals, and the US Government Principals regarding the care and use of animals. All protocols involving mice were approved under the animal proposal number 2010-0053 (05/06/2010). The mice were housed in the specific pathogen free facility on the North campus of UT Southwestern Medical Center.

### Mice

All mice used were on a C57BL/6 background. CD3 epsilon deficient (CD3ε^−/−^) mice were provided by Dr. Paul Love of the NIH [61]. The mice were housed in a Specific Pathogen Free facility on the North Campus of the University of Texas Southwestern Medical Center (Dallas, TX). Lipopolysaccharide (LPS) and dexamethasone (Dex) were purchased from Sigma Chemical Co (St. Louis, MO) and prepared at 1 mg/ml and 0.06 mg/ml (active component, 1.5×10^−4^ M stock) in water, respectively. Unless otherwise indicated, male mice of 5–8 weeks of age were used in all the experiments involving injections with PBS, LPS, or Dex.

### Luciferase Reporter Assays

PCR-amplified genetic fragments (500–700 base pairs) containing miR-181a or miR-181d were cloned into the pCDNA3.1 vector (Invitrogen, Carlsbad, CA). The 3′ untranslated regions of murine Cd69, Prox1, and Lif genes were amplified by PCR and ligated into the firefly luciferase reporter construct (pMIR-REPORT, Ambion, Austin, TX). Primers sequences are provided in Figure S4. These constructs were co-transfected in COS-7 cells in combination with a beta-galactosidase expression vector, using the Fugene 6 Transfection Reagent according to the manufacturer's instructions (Roche, Indianapolis, IN). The total amount of DNA per well was kept equivalent by adding the corresponding amount of empty vector (pCDNA3.1). Forty-eight hours after transfection, cells were harvested and analyzed for luciferase expression using the Luciferase Assay Kit (Promega, Madison, WI). Relative luciferase activity determined by the ratio of the firefly luciferase normalized to the beta-galactosidase expression levels for each of the transfectants. All transfections were performed in triplicate, and the experiments were performed on at least 3 separate occasions.

### Cell Isolation and Analyses

Lymphocytes were isolated from the thymus and spleen as previously described [62]. FACS™ staining buffer consisted of 1% fetal calf serum in PBS containing Ca^2+^ and Mg^2+^ (Mediatech, Herndon, VA). Between 1–2.5×10^6^ cells were pre-treated for 10–30 minutes at 4°C with the 2.4G2 mAb to block non-specific Fc receptor binding. Cells were then stained with the various antibodies for 30 minutes at 4°C followed by washing and analyses on flow cytometers. Ten thousand to 1×10^6^ cells per sample were acquired on a FACSCalibur (BD Biosciences, San Jose, CA). Data was analyzed using FlowJo software (Tree Star, Inc., Ashland, OR). For isolating thymocyte subsets, 10^8^ thymocytes from control- (PBS) and LPS-treated mice were stained with anti-CD4-PE and anti-CD8-FITC labeled mAbs, followed by high-speed cell sorting on a Mo-Flo (Cytomation). One hundred thousand CD4^−^CD8^−^ (DN), CD4^+^CD8^+^ (DP), CD4^+^CD8^−^ (CD4 SP), and CD4^−^CD8^+^ (CD8 SP) thymocytes were sorted to >95% purity, lysed in 1.0 ml of Trizol, and processed for total RNA as described below. In certain experiments, single cell suspensions were prepared using thymocytes from the control and LPS-treated mice. T cells were enriched to greater than 95% purity from these cell suspensions with the EasySep T cell Enrichment kits following the manufacturers' guidelines (StemCell Technologies, Vancouver, Canada). The brain, heart, and kidney were isolated using standard protocols, and a small extract was used to isolate RNA. This was performed in Trizol followed by Dounce homogenization.

### RNA Analysis

Total RNA (including microRNA) was isolated from purified T cells, thymocyte subsets, or tissue samples obtained from control and treated mice by using either standard Trizol extraction procedures or the miRNeasy kit (Qiagen Inc., Valencia, CA), based on the manufacturer's instructions, respectively. RNA quality was confirmed with an absorbance ratio at UV_260/280_ greater than 1.80. For northern blotting procedures, 5 µg of total RNA was resolved on 15% urea/polyacrylamide gels and transferred to Zeta probe membranes. Similar amounts of RNA were loaded onto the gels, as assessed by ethidium bromide staining prior to electrophoretic transfer to the membrane. Following UV-cross linking, the membranes were probed with selected miR probes labeled with [^32^P]-dATP. The miR probes contained a poly-A tail for radiolabeling under conditions suggested by the manufacturer (Starfire kit, Idt DNA Technologies, Coralville, Iowa). Blots were exposed and quantified by using a phosphorimager and ImageQuant (Bio-Rad Laboratories, Hercules, CA). Detection of U6 levels were used an endogenous controls to normalize total RNA input. For analysis of miR expression levels in thymocyte subsets by real-time microRNA PCR, total RNA was isolated using miRNeasy and DNase-digested (Turbo-DNAse, Ambion). cDNA was made from 10 ng of total RNA using the TaqMan MicroRNA Reverse Transcription Kit (Applied Biosystems, Carlsbad, CA). Real-time PCR analysis was performed using TaqMan Gene Expression Master Mix and microRNA-specific TaqMan probes in the microRNA assays on an ABI 7300 series PCR machine (Applied Biosystems) according to the manufacturers' recommendations. U6 small RNA (Applied Biosystems) was used as an endogenous control for all miRs. All reactions were performed in triplicate, and repeated at least three times. Relative expression of miRs was calculated by the comparative threshold method (ΔΔC_T_). Student t-tailed tests were performed using comparisons with a defined relative threshold set at 1.

### MicroRNA arrays

MicroRNA arrays were from LC Sciences (LS Sciences, Houston, TX). In brief, 6 RNA samples (5 µg of total thymic RNA per sample) were prepared from 3 control (PBS) and 3 LPS-treated C57Bl/6 male mice of 6–8 weeks of age. These were sent to LC Sciences for microRNA profiling. The RNA was labeled with Cy3 (2 control mice and 1 LPS-injected mouse) or Cy5 (2 LPS injected mice and 1 control mouse). The various labeled samples were analyzed on the microarray platform with a probe set containing 649 murine miRs, based on the Sanger Version 12.0 and 13.0 database release (Data Set S1).

### Statistical analyses

In the LPS and Dex treatments, one-way ANOVA was used to determine the significance. For all other assays, unpaired Student's t-test was used for determining the significance. All data with significant differences were indicated by one asterisk (p<0.05), two asterisks (p<0.01), or three asterisks (p<0.001).

## Supporting Information

Figure S1 **Lipopolysaccharide and dexamethasone have differential effects on peripheral lymphocytes.** A) Lymphocytes were isolated from control (PBS injected, t = 72 h), LPS, or Dex injected mice (t = 24, 48 and 72 h). The total lymphoid cellularity was determined. B–D) The splenic cells were stained with fluorochrome-labeled anti-B220, anti-CD3, anti-CD4, and anti-CD8 mAbs and analyzed by flow cytometry. The absolute number of B) B220^+^ B cells, C) CD4^+^CD8^−^ T cells, and D) CD4^−^CD8^+^ T cells were calculated at 24, 48, and 72 h post-injection, after appropriate electronic gating to determine the percentage of each population. Data are representative of mean +/− SD from at least 5 mice per group (*p<0.05, **p<0.01, *** p<0.001 versus PBS control; one-way ANOVA analyses. E) Representative heat map shows differential expression of miRNAs in the thymus of control (PBS) and LPS-treated mice (t = 72 h). The samples were labeled with Cy3 and Cy5, respectively, and used to probe a murine microRNA array containing 649 miRs (LC Sciences). Red indicates high miR expression; green indicates low miR expression in thymic tissue. Data are shown for one of three separate microarray analyses with control and LPS injected mice.
(TIF)

Figure S2 **MiR expression patterns in different tissues.** A) Differential expression of microRNAs in the thymic tissue. RNA was prepared from the thymus of age- and sex- matched control and CD3ε^−/−^ C57BL/6 mice. The samples were used to probe a murine microRNA array containing 649 miRs (LC Sciences). Data represent differential expression levels of selected miRs from two independent sample preparations. B) Differential expression of stress responsive miRs in diverse tissues. Male mice were injected with PBS, LPS or Dex. Total RNA was isolated from the heart, kidney, and liver of control (PBS) and LPS- or Dex- treated mice at 72 h post-injection. The individual miRs (miR-150, miR-205, miR-128, miR-181a, miR-181b, miR-181d) were detected by Northern blotting. The relative amounts of a control RNA were determined by blotting for U6. C) Male mice were injected with PBS, LPS or Dex. Total RNA was isolated from the heart, kidney, liver, brain, spleen, and thymus of control (PBS) and LPS- or Dex- treated mice at 72 h post-injection. The individual miRs (miR-15a, miR-17, miR-20a, miR-20b, miR-26b, miR-106a, miR-125-5p, miR-342-3p) were detected by Northern blotting. The relative amounts of a control RNA were determined by blotting for U6.
(TIF)

Figure S3 **Stress responsive changes in thymic miR profiles are time dependent.** A) Total RNA was isolated from thymic tissue prepared from PBS- (lane 1), LPS- (lanes 2–4), and Dex-treated (5–7) mice at 24 h (lanes 2, 5), 48 h (lanes 3, 6), and 72 h (lanes 1, 4, 7). In lanes 8–9, T cell were purified from the thymus preparation prior to Northern blotting for the selected miRs. The samples were quantified by phoshoimager analyses, following background subtraction, and controlling for total RNA amounts with a U6 probe. Data shown is mean +/1 SD of relative fold changes in miR expression levels of PBS- versus LPS- or Dex-treated samples from 3–5 independent northern blots.
(TIF)

Figure S4 **MiR-181 interaction sites within murine LIF.** A) Predicted interactions of miR-181a and miR-181d with their binding sites in the murine LIF 3′UTR. RNAhybrid algorithm and the microRNA resource (www.microrna.org) were used to assess potential miR binding target sites. B) List of primers used for PCR amplification of miR-181a, miR-181d, and the Lif 3′ UTR. C) Mutation of the Lif sequences at site 1 and site 572 are shown. The seed sequence of miR-181 is underlined.
(TIF)

Figure S5 **Dose-response analysis of miR-181 target genes.** A beta-galactosidase expressing vector and the luciferase reporter constructs containing the 3′untranslated region of murine Cd69, Prox1, and Lif genes were co-transfected along with vector alone or vectors expressing miR-181a or miR-181d at the indicated amounts (100 or 300 ng). Firefly luciferase was normalized to beta-galactosidase activity. Each graph represents mean +/− SD, using the ratio of the normalized luciferase activity in miR-181 and control vector transfections. This was done in three independent experiments, with each sample tested in triplicate or quadruplicate (n.s., not significant, * p<0.05, ** p<0.01, *** p<0.001 versus vector control, unpaired Student's t-test).
(TIF)

Data Set S1 **Standard Data Analysis Report.**
(PDF)
