# Supplementary figures and images for: Dynamic Modulation of Thymic MicroRNAs in Response to Stress
Source: PLoS One. 2011 Nov 16;6(11):e27580. doi: 10.1371/journal.pone.0027580 (PMC3217971; doi:10.1371/journal.pone.0027580)

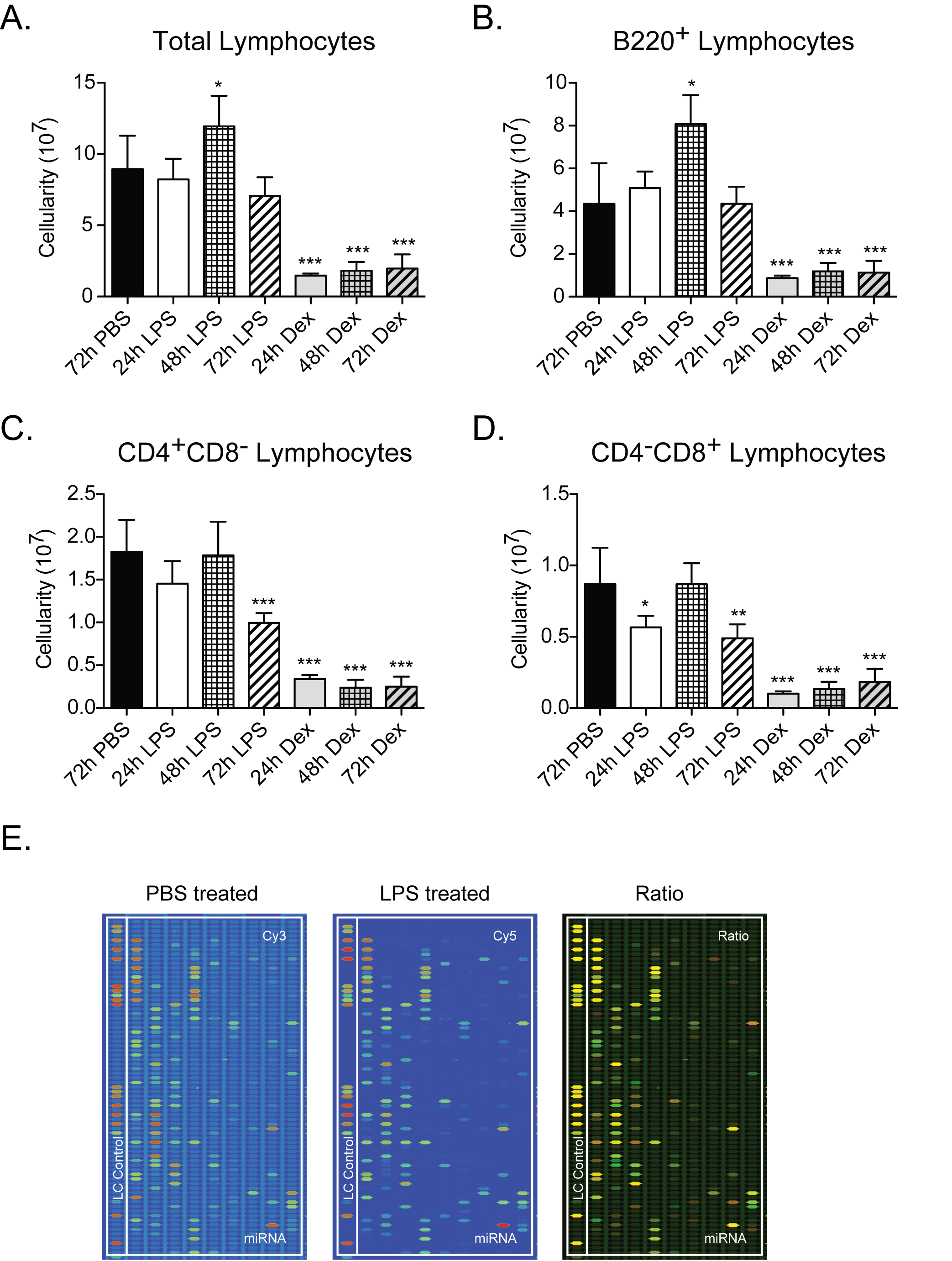

Supplement: Figure S1 — Lipopolysaccharide and dexamethasone have differential effects on peripheral lymphocytes. A) Lymphocytes were isolated from control (PBS injected, t = 72 h), LPS, or Dex injected mice (t = 24, 48 and 72 h). The total lymphoid cellularity was determined. B–D) The splenic cells were stained with fluorochrome-labeled anti-B220, anti-CD3, anti-CD4, and anti-CD8 mAbs and analyzed by flow cytometry. The absolute number of B) B220+ B cells, C) CD4+CD8− T cells, and D) CD4−CD8+ T cells were calculated at 24, 48, and 72 h post-injection, after appropriate electronic gating to determine the percentage of each population. Data are representative of mean +/− SD from at least 5 mice per group (*p<0.05, **p<0.01, *** p<0.001 versus PBS control; one-way ANOVA analyses. E) Representative heat map shows differential expression of miRNAs in the thymus of control (PBS) and LPS-treated mice (t = 72 h). The samples were labeled with Cy3 and Cy5, respectively, and used to probe a murine microRNA array containing 649 miRs (LC Sciences). Red indicates high miR expression; green indicates low miR expression in thymic tissue. Data are shown for one of three separate microarray analyses with control and LPS injected mice. (TIF) [file pone.0027580.s001.tif]

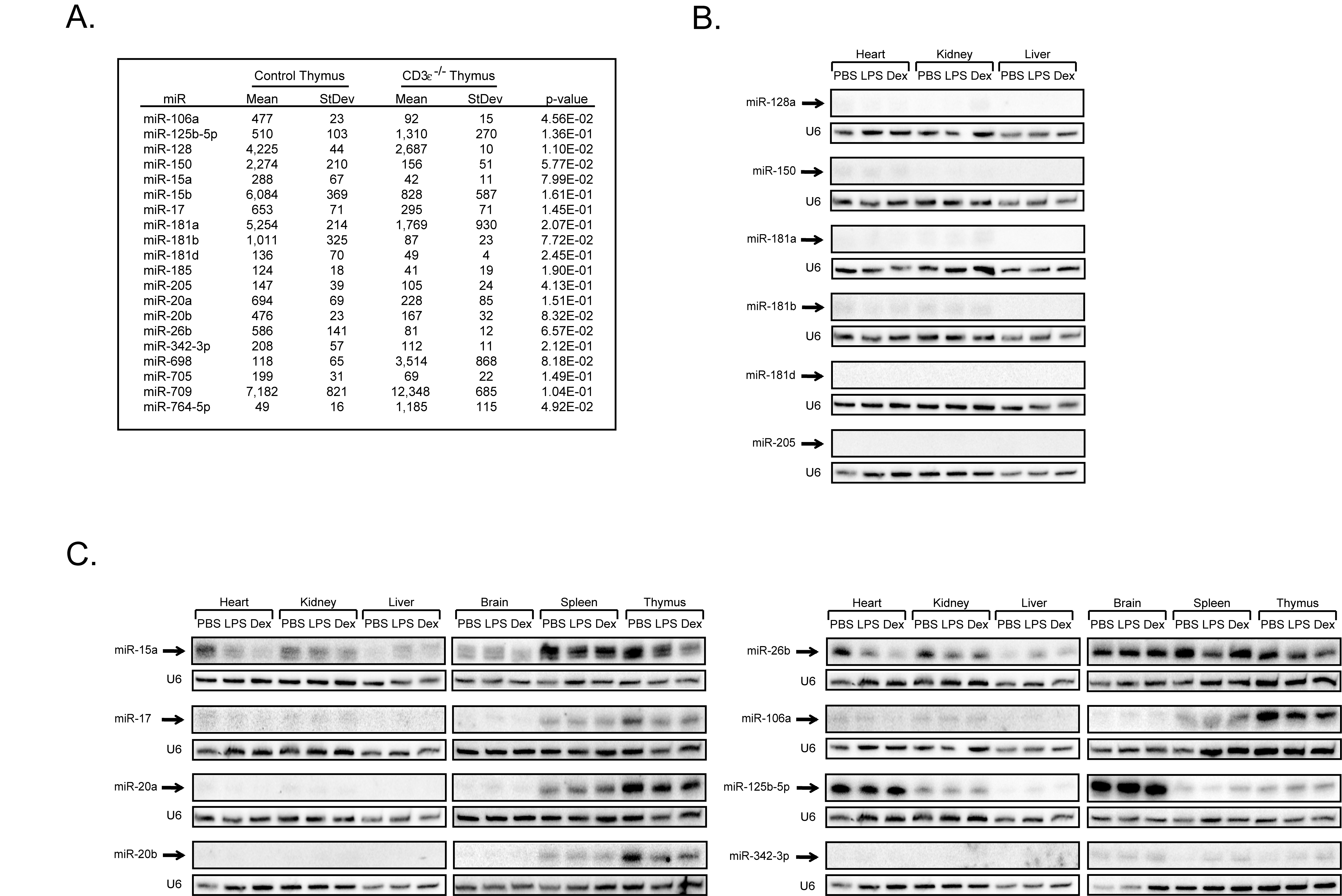

Supplement: Figure S2 — MiR expression patterns in different tissues. A) Differential expression of microRNAs in the thymic tissue. RNA was prepared from the thymus of age- and sex- matched control and CD3ε−/− C57BL/6 mice. The samples were used to probe a murine microRNA array containing 649 miRs (LC Sciences). Data represent differential expression levels of selected miRs from two independent sample preparations. B) Differential expression of stress responsive miRs in diverse tissues. Male mice were injected with PBS, LPS or Dex. Total RNA was isolated from the heart, kidney, and liver of control (PBS) and LPS- or Dex- treated mice at 72 h post-injection. The individual miRs (miR-150, miR-205, miR-128, miR-181a, miR-181b, miR-181d) were detected by Northern blotting. The relative amounts of a control RNA were determined by blotting for U6. C) Male mice were injected with PBS, LPS or Dex. Total RNA was isolated from the heart, kidney, liver, brain, spleen, and thymus of control (PBS) and LPS- or Dex- treated mice at 72 h post-injection. The individual miRs (miR-15a, miR-17, miR-20a, miR-20b, miR-26b, miR-106a, miR-125-5p, miR-342-3p) were detected by Northern blotting. The relative amounts of a control RNA were determined by blotting for U6. (TIF) [file pone.0027580.s002.tif]

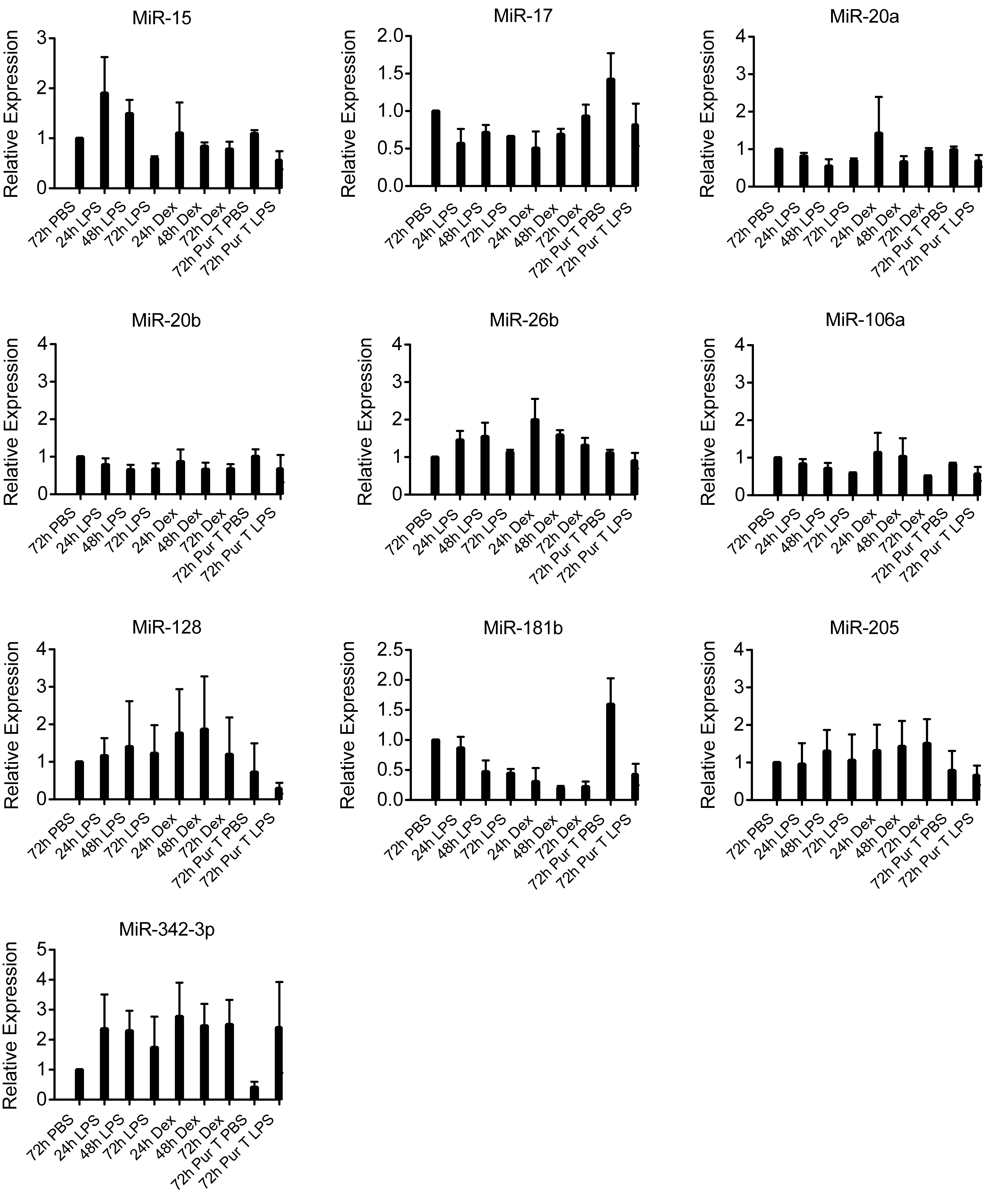

Supplement: Figure S3 — Stress responsive changes in thymic miR profiles are time dependent. A) Total RNA was isolated from thymic tissue prepared from PBS- (lane 1), LPS- (lanes 2–4), and Dex-treated (5–7) mice at 24 h (lanes 2, 5), 48 h (lanes 3, 6), and 72 h (lanes 1, 4, 7). In lanes 8–9, T cell were purified from the thymus preparation prior to Northern blotting for the selected miRs. The samples were quantified by phoshoimager analyses, following background subtraction, and controlling for total RNA amounts with a U6 probe. Data shown is mean +/1 SD of relative fold changes in miR expression levels of PBS- versus LPS- or Dex-treated samples from 3–5 independent northern blots. (TIF) [file pone.0027580.s003.tif]

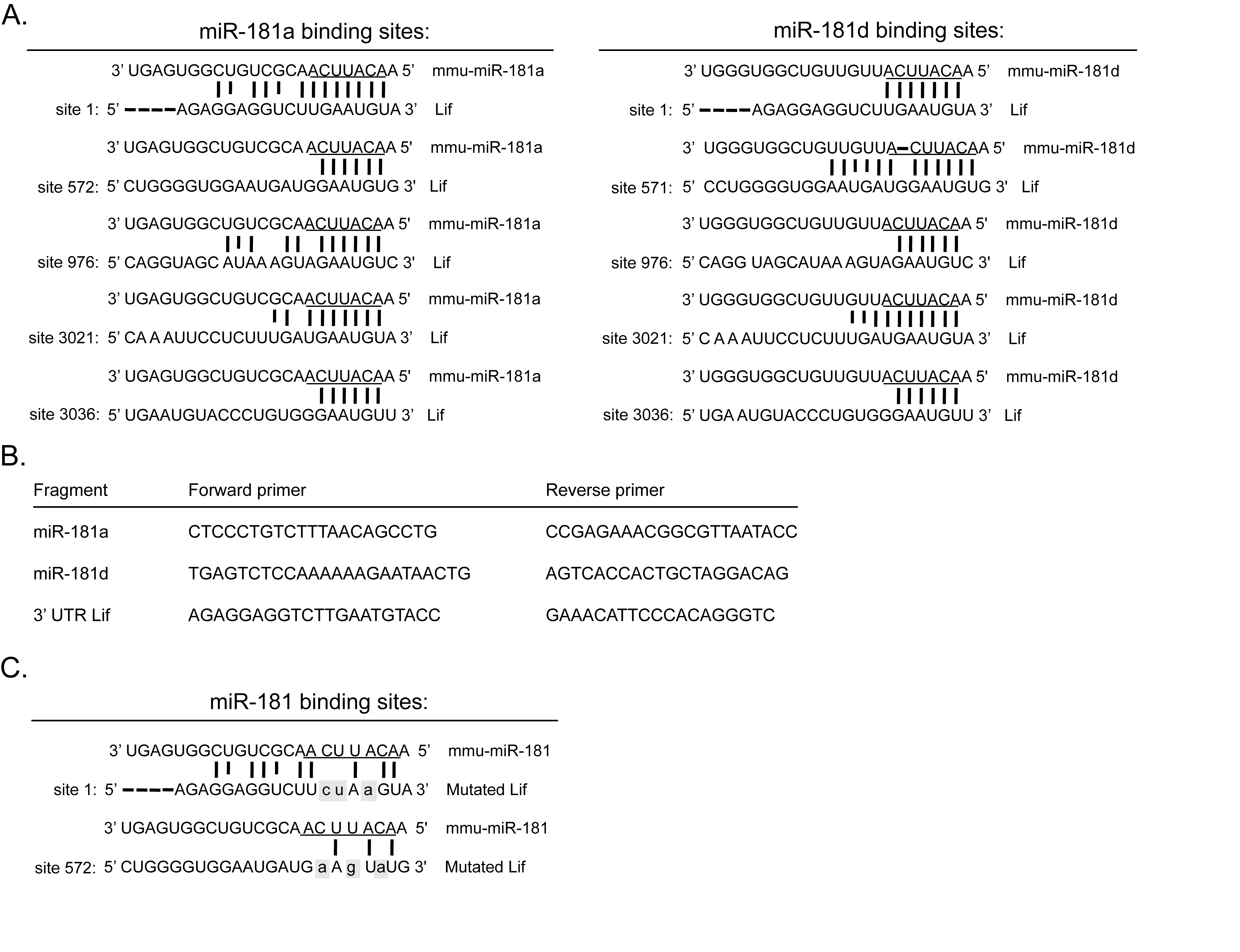

Supplement: Figure S4 — MiR-181 interaction sites within murine LIF. A) Predicted interactions of miR-181a and miR-181d with their binding sites in the murine LIF 3′UTR. RNAhybrid algorithm and the microRNA resource (www.microrna.org) were used to assess potential miR binding target sites. B) List of primers used for PCR amplification of miR-181a, miR-181d, and the Lif 3′ UTR. C) Mutation of the Lif sequences at site 1 and site 572 are shown. The seed sequence of miR-181 is underlined. (TIF) [file pone.0027580.s004.tif]

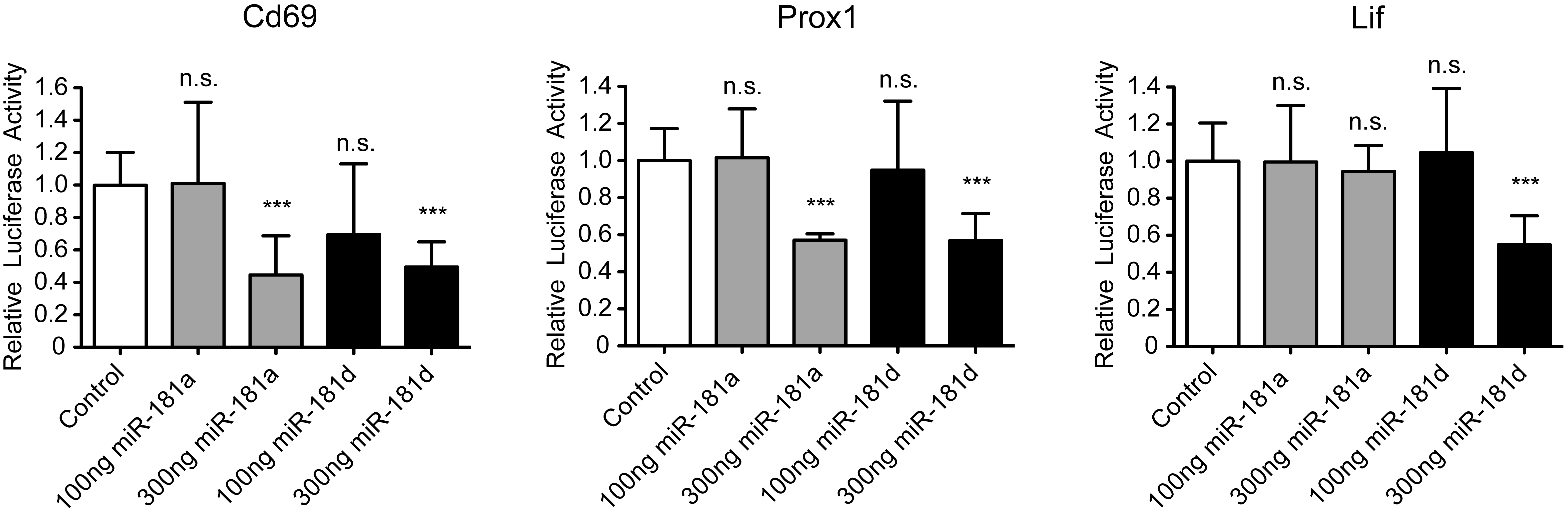

Supplement: Figure S5 — Dose-response analysis of miR-181 target genes. A beta-galactosidase expressing vector and the luciferase reporter constructs containing the 3′untranslated region of murine Cd69, Prox1, and Lif genes were co-transfected along with vector alone or vectors expressing miR-181a or miR-181d at the indicated amounts (100 or 300 ng). Firefly luciferase was normalized to beta-galactosidase activity. Each graph represents mean +/− SD, using the ratio of the normalized luciferase activity in miR-181 and control vector transfections. This was done in three independent experiments, with each sample tested in triplicate or quadruplicate (n.s., not significant, * p<0.05, ** p<0.01, *** p<0.001 versus vector control, unpaired Student's t-test). (TIF) [file pone.0027580.s005.tif]
