# Supplementary material for: Dynamic Modulation of Thymic MicroRNAs in Response to Stress
Source: PLoS One. 2011 Nov 16;6(11):e27580. doi: 10.1371/journal.pone.0027580 (PMC3217971; doi:10.1371/journal.pone.0027580)
Supplement: Data Set S1 — Standard Data Analysis Report. (PDF) [file pone.0027580.s006.pdf]

Standard Data Analysis Report
miRNA Microarray Service

Experiment: S81780
Date: 2009-04-10
Prepared for: Dr. Nicolai S.C. van Oers
UT Southwestern Medical Center

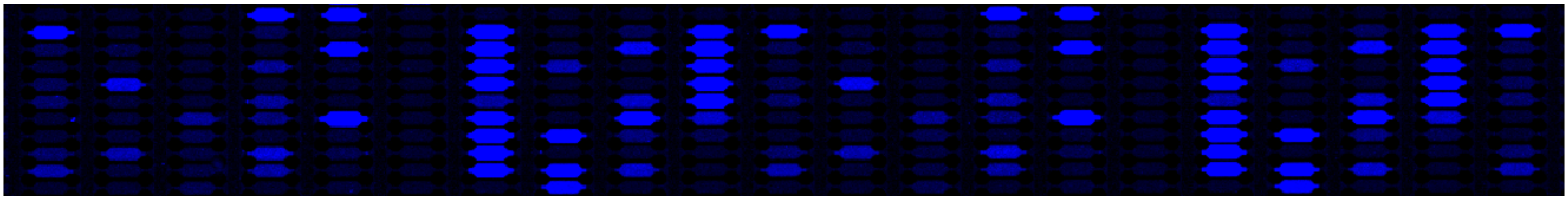

Prepared by
LC Sciences, LLC | **www.LCsciences.com** | **support@LCsciences.com**
2575 W. Bellfort, Suite 270, Houston, Texas 77054
Tel. 713 664-7087, Fax 713 664-8181

I. Data List

Chip 1

**Data Files**	
Folder Name	Chip01_M12.0_090176
Data File	01_M12.0_090176-0408-Oers-Ctrl1+LPS1-425-500_Data.xls
Layout File	MiRMouse_12.0_080901.xls
Original Image File	01_M12.0_090176-0408-Oers-Ctrl1+LPS1-425-500.tif
Processed Cy3 File	01_M12.0_090176-0408-Oers-Ctrl1+LPS1-425cy3.tif
Processed Cy5 File	01_M12.0_090176-0408-Oers-Ctrl1+LPS1-500cy5.tif
**Assay Information**	
Date of Assay	2009-4-8
Chip ID	01_M12.0_090176
Sample source	UT Southwestern Medical Center
Sample A	
Sample ID	Thymus-Ctl-1
Sample Receiving Date	2009-1-26
Labeling Dye	Cy3
Sample B	
Sample ID	Thymus-LPS-1
Sample Receiving Date	2009-1-26
Labeling Dye	Cy5

Chip 2

**Data Files**	
Folder Name	Chip02_M12.0_090363
Data File	02_M12.0_090363-0408-Oers-Ctrl2+LPS2-430-480_Data.xls
Layout File	MiRMouse_12.0_080901.xls
Original Image File	02_M12.0_090363-0408-Oers-Ctrl2+LPS2-430-480.tif
Processed Cy3 File	02_M12.0_090363-0408-Oers-Ctrl2+LPS2-430cy3.tif
Processed Cy5 File	02_M12.0_090363-0408-Oers-Ctrl2+LPS2-480cy5.tif
**Assay Information**	
Date of Assay	2009-4-8
Chip ID	02_M12.0_090363
Sample source	UT Southwestern Medical Center
Sample A	
Sample ID	Thymus-Ctl-2
Sample Receiving Date	2009-1-26
Labeling Dye	Cy3
Sample B	
Sample ID	Thymus-LPS-2
Sample Receiving Date	2009-1-26
Labeling Dye	Cy5

Chip 3

Data Files	
Folder Name	Chip03_M12.0_090444
Data File	03_M12.0_090444-0408-Oers-LPS3+Ctrl3-430-450_Data.xls
Layout File	MiRMouse_12.0_080901.xls
Original Image File	03_M12.0_090444-0408-Oers-LPS3+Ctrl3-430-450.tif
Processed Cy3 File	03_M12.0_090444-0408-Oers-LPS3+Ctrl3-430cy3.tif
Processed Cy5 File	03_M12.0_090444-0408-Oers-LPS3+Ctrl3-450cy5.tif
**Assay Information**	
Date of Assay	2009-4-8
Chip ID	03_M12.0_090444
Sample source	UT Southwestern Medical Center
Sample A	
Sample ID	Thymus-LPS-3
Sample Receiving Date	2009-1-26
Labeling Dye	Cy3
Sample B	
Sample ID	Thymus-Ctl-3
Sample Receiving Date	2009-1-26
Labeling Dye	Cy5

II. Chip Content

The content of each chip is listed in a Layout File of a corresponding folder. Multiple redundant regions are included. Each region further comprises a miRNA probe region, which detects miRNA transcripts listed in Sanger miRBase Release 12.0 (http://www.sanger.ac.uk/Software/Rfam/mirna/).

Multiple control probes are included in each chip. The control probes are used for quality controls of chip production, sample labeling and assay conditions. Among the control probes, PUC2PM-20B and PUC2MM-20B are the perfect match and single-based match detection probes, respectively, of a 20-mer RNA positive control sequence that is spiked into the RNA samples before labeling. One may assess assay stringency from the intensity ratio of PUC2PM-20B and PUC2MM-20B, which is normally larger than 30.

When the option for custom probes is selected, custom probes are also included.

III. Summary of Results

Following are representative regions of chips images. From Cy3 and Cy5 images one may directly read miRNA profiles and from Ratio images one may get a quick sense of differential expressions between the corresponding samples. The images are displayed in pseudo colors so as to expand visual dynamic range. In the Cy3 and Cy5 intensity images, as signal intensity increases from 1 to 65,535 the corresponding color changes from blue to green, to yellow, and to red. In the Cy3/Cy5 ratio image, when Cy3 level is higher than Cy5 level the color is green; when Cy3 level is equal to Cy5 level the color is yellow; and when Cy5 level is higher than Cy3 level the color is red.

In this section, a list of differentially expressed transcripts is also provided following the chip images. From the list, one can have a quick overview of the difference between the two samples on the chip.

Chip: 01_M12.0_090176

Sample A: Thymus-Ctl-1 – Cy3
Sample B: Thymus-LPS-1 – Cy5

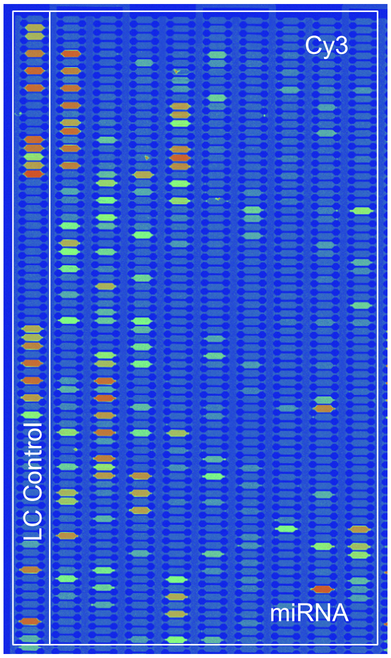

Sample A

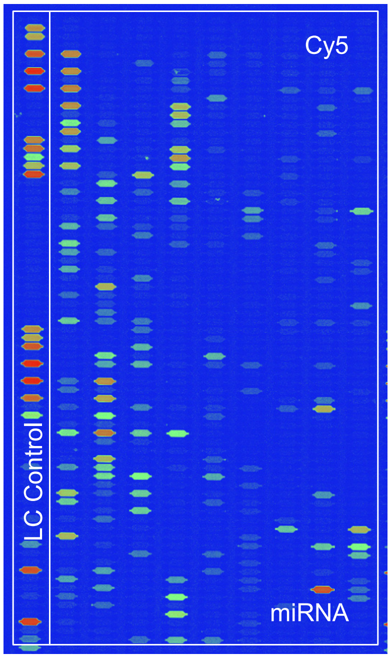

Sample B

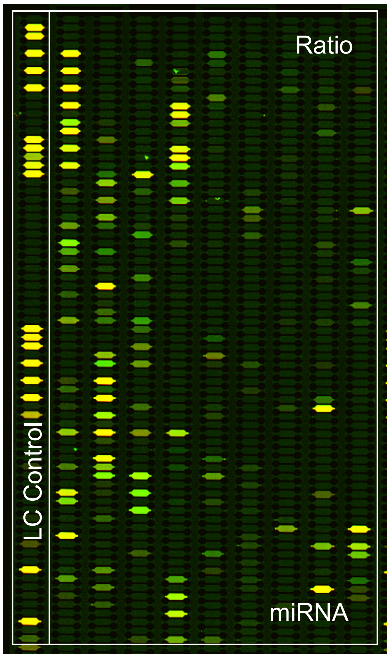

Cy3/Cy5

Table 1 Call list (differentially expressed transcripts with p-value < 0.01)

No.	Probe_ID	Sample A Signal	Sample B Signal	log2 (Sample B / Sample A)
1	mmu-miR-29b	296.66	46.05	-2.68
2	mmu-miR-301a	228.77	39.42	-2.31
3	mmu-miR-340-5p	787.34	159.28	-2.29
4	mmu-miR-101a	184.18	37.52	-2.25
5	mmu-miR-29c	448.51	94.00	-2.14
6	mmu-miR-142-5p	1,235.82	326.59	-1.90
7	mmu-miR-15b*	891.44	252.07	-1.89
8	mmu-miR-7a	1,042.42	282.43	-1.82
9	mmu-miR-98	1,571.08	465.49	-1.72
10	mmu-miR-148a	2,491.06	720.40	-1.71
11	mmu-miR-322	207.60	62.79	-1.67
12	mmu-miR-685	174.83	505.92	1.64
13	mmu-miR-720	450.13	1,308.59	1.56
14	mmu-miR-15a	10,292.09	3,602.68	-1.51
15	mmu-miR-19b	3,786.06	1,279.15	-1.50
16	mmu-miR-195	3,644.37	1,316.02	-1.49
17	mmu-miR-30e*	333.08	134.58	-1.37
18	mmu-miR-99b	738.44	1,799.12	1.32
19	mmu-miR-1195	572.87	1,370.85	1.32
20	mmu-miR-699	1,156.76	2,815.97	1.28
21	mmu-miR-30e	1,490.27	597.84	-1.27
22	mmu-miR-350	1,460.70	626.10	-1.22
23	mmu-miR-26b	17,410.80	7,883.38	-1.17
24	mmu-miR-872	347.31	159.89	-1.13
25	mmu-miR-20b	14,923.96	6,734.18	-1.12
26	mmu-miR-181d	17,213.20	8,074.17	-1.11
27	mmu-miR-342-3p	2,383.74	5,149.05	1.11
28	mmu-miR-130a	283.81	127.91	-1.11
29	mmu-miR-342-5p	150.68	334.84	1.09
30	mmu-miR-99a	571.96	1,198.36	1.06
31	mmu-miR-455	233.46	484.92	1.06
32	mmu-miR-374	2,135.47	1,035.72	-1.04
33	mmu-miR-21	15,702.73	7,808.99	-1.04
34	mmu-miR-375	398.40	805.32	1.01
35	mmu-miR-100	846.17	1,527.40	0.97
36	mmu-miR-150	12,916.80	25,390.26	0.97
37	mmu-miR-205	3,509.09	6,733.07	0.94
38	mmu-miR-27a	5,057.84	2,741.28	-0.92
39	mmu-miR-652	239.38	443.18	0.90
40	mmu-miR-222	843.14	1,520.02	0.88
41	mmu-miR-223	533.56	296.87	-0.85
42	mmu-miR-146b	1,196.31	672.63	-0.84
43	mmu-let-7e	12,572.12	6,255.07	-0.84
44	mmu-miR-425	1,050.71	1,886.38	0.83
45	mmu-miR-106a	12,884.05	7,272.62	-0.80
46	mmu-miR-106b	4,741.74	2,959.81	-0.76

47	mmu-miR-20a	18,186.18	10,530.52	-0.74
48	mmu-miR-125b-5p	13,356.56	21,590.30	0.71
49	mmu-miR-709	30,745.89	49,634.91	0.70
50	mmu-miR-17	16,569.94	10,339.30	-0.69
51	mmu-miR-22	604.75	983.28	0.68
52	mmu-miR-30a	4,583.82	2,904.82	-0.66
53	mmu-miR-152	1,995.19	1,332.82	-0.58
54	mmu-miR-200b	3,125.05	4,648.78	0.57
55	mmu-miR-574-5p	4,995.90	7,621.59	0.56
56	mmu-miR-199a-3p	2,590.65	1,809.05	-0.55
57	mmu-miR-140*	1,931.32	2,662.55	0.55
58	mmu-miR-181a	25,045.15	34,375.14	0.53
59	mmu-miR-214	1,202.33	1,851.28	0.53
60	mmu-miR-689	1,617.13	1,179.50	-0.52
61	mmu-miR-1187	4,810.75	6,680.76	0.49
62	mmu-let-7g	21,805.46	14,934.60	-0.49
63	mmu-miR-191	14,114.24	19,347.92	0.48
64	mmu-miR-145	4,135.73	5,638.22	0.48
65	mmu-let-7b	21,623.09	29,929.49	0.47
66	mmu-miR-126-3p	10,122.41	6,999.85	-0.46
67	mmu-miR-92a	17,743.54	24,704.99	0.46
68	mmu-miR-27b	7,537.86	5,503.23	-0.45
69	mmu-miR-200a	1,443.17	1,081.89	-0.42
70	mmu-miR-423-5p	2,313.28	3,114.85	0.41
71	mmu-let-7c	24,423.95	33,513.30	0.40
72	mmu-miR-378	1,319.89	1,716.17	0.40
73	mmu-miR-23b	15,951.74	20,393.04	0.38
74	mmu-miR-155	6,545.02	8,905.57	0.38
75	mmu-miR-130b	4,106.66	3,046.42	-0.38
76	mmu-miR-23a	15,975.01	19,174.42	0.33
77	mmu-miR-93	6,632.66	5,295.45	-0.33
78	mmu-miR-200c	3,765.86	4,651.03	0.33
79	mmu-miR-30b	14,844.92	11,783.94	-0.32
80	mmu-miR-15b	25,865.73	31,998.07	0.31
81	mmu-miR-1224	8,085.13	9,887.74	0.29
82	mmu-let-7d	23,869.65	29,395.12	0.27
83	mmu-let-7i	21,191.88	16,701.04	-0.27
84	mmu-miR-762	6,345.34	7,396.67	0.26
85	mmu-miR-92b	10,188.80	12,153.71	0.24
86	mmu-miR-690	20,314.13	22,958.42	0.23
87	mmu-miR-26a	25,848.47	29,857.31	0.21
88	mmu-miR-30c	13,982.24	16,279.50	0.16
89	mmu-miR-128	21,298.50	23,762.39	0.14

Chip: 02_M12.0_090363

Sample A: Thymus-Ctl-2 – Cy3
Sample B: Thymus-LPS-2 – Cy5

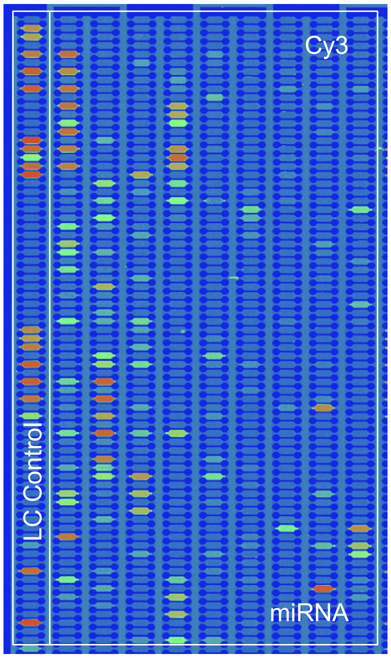

Sample A

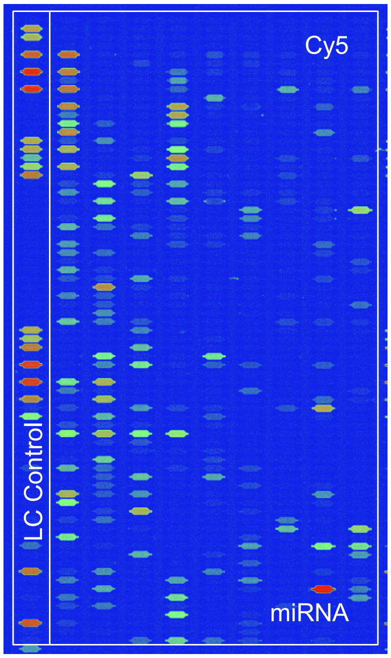

Sample B

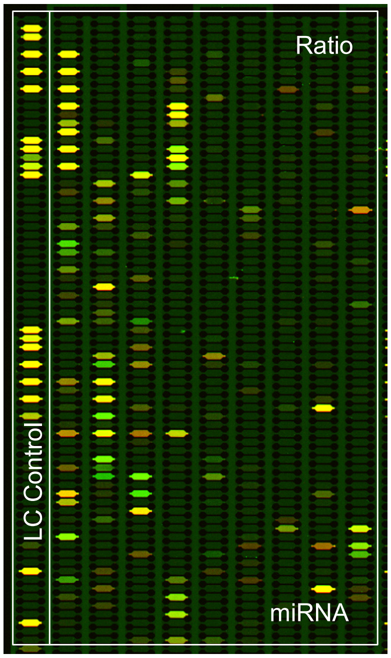

Cy3/Cy5

Table 2 Call list (differentially expressed transcripts with p-value < 0.01)

No.	Probe_ID	Sample A Signal	Sample B Signal	log2 (Sample B / Sample A)
1	mmu-miR-181d	7,277.67	348.58	-4.30
2	mmu-miR-680	11.48	188.62	4.09
3	mmu-miR-19b	3,207.98	279.77	-3.49
4	mmu-miR-374	1,037.18	90.25	-3.38
5	mmu-let-7d*	96.78	987.99	3.35
6	mmu-miR-290-5p	31.19	309.11	3.23
7	mmu-miR-124	109.48	11.09	-3.21
8	mmu-miR-15b*	430.79	47.97	-3.06
9	mmu-miR-1	27.74	170.25	2.81
10	mmu-miR-20b	11,968.87	1,684.13	-2.78
11	mmu-miR-466i	381.13	2,126.80	2.69
12	mmu-miR-15a	8,698.07	1,445.19	-2.59
13	mmu-miR-106a	10,206.72	1,705.58	-2.58
14	mmu-miR-18a	1,069.33	174.07	-2.55
15	mmu-miR-574-3p	226.88	1,344.70	2.51
16	mmu-miR-139-5p	62.32	336.84	2.47
17	mmu-miR-466f-3p	517.82	2,809.29	2.42
18	mmu-miR-669f	232.15	1,210.60	2.42
19	mmu-miR-181c	2,996.18	585.71	-2.37
20	mmu-miR-350	1,065.69	211.53	-2.33
21	mmu-miR-99a	290.10	1,417.36	2.31
22	mmu-miR-363	280.35	52.79	-2.31
23	mmu-miR-467f	616.18	2,899.41	2.23
24	mmu-miR-705	2,170.69	9,326.98	2.17
25	mmu-miR-375	315.04	1,346.87	2.07
26	mmu-miR-130b	4,482.86	1,006.62	-2.06
27	mmu-miR-181b	21,790.63	5,287.32	-2.04
28	mmu-miR-206	78.47	367.20	1.97
29	mmu-miR-340-5p	217.61	49.15	-1.96
30	mmu-miR-17	14,781.62	3,603.93	-1.95
31	mmu-miR-20a	17,180.29	4,553.57	-1.92
32	mmu-miR-467b*	85.03	330.04	1.91
33	mmu-miR-10a	89.11	311.34	1.91
34	mmu-miR-181a-1*	399.56	103.78	-1.90
35	mmu-miR-106b	4,887.41	1,314.91	-1.88
36	mmu-miR-205	2,494.01	8,657.20	1.82
37	mmu-miR-762	4,170.03	14,604.68	1.80
38	mmu-miR-421	221.71	60.52	-1.77
39	mmu-miR-214	1,232.22	3,998.55	1.74
40	mmu-miR-93	6,869.10	2,025.74	-1.71
41	mmu-miR-1224	3,867.48	12,519.90	1.65
42	mmu-miR-30e	748.33	240.00	-1.64
43	mmu-miR-100	478.93	1,463.82	1.63
44	mmu-miR-99b	765.83	2,353.29	1.58
45	mmu-miR-128	23,061.81	7,504.53	-1.58
46	mmu-miR-466g	176.37	527.31	1.57

47	mmu-miR-122	115.96	329.98	1.56
48	mmu-miR-805	2,161.89	764.44	-1.48
49	mmu-miR-125a-5p	1,660.09	4,671.76	1.46
50	mmu-miR-1892	562.78	1,517.38	1.43
51	mmu-miR-455	316.62	750.98	1.33
52	mmu-miR-10b	507.23	1,251.31	1.30
53	mmu-miR-744	130.80	309.52	1.24
54	mmu-miR-200b	1,869.13	4,251.20	1.23
55	mmu-miR-709	29,608.85	65,078.30	1.23
56	mmu-miR-150	13,438.50	30,252.61	1.23
57	mmu-miR-1195	3,724.27	8,715.82	1.23
58	mmu-miR-98	383.88	168.88	-1.22
59	mmu-miR-148a	1,541.69	639.02	-1.22
60	mmu-miR-125b-5p	10,318.68	23,984.35	1.20
61	mmu-miR-185	1,769.65	756.85	-1.20
62	mmu-miR-1894-3p	1,391.57	3,174.18	1.18
63	mmu-miR-182	168.22	362.16	1.12
64	mmu-miR-199a-3p	1,414.16	2,950.07	1.06
65	mmu-miR-429	474.74	957.48	1.05
66	mmu-miR-342-3p	3,353.42	6,846.13	1.03
67	mmu-miR-532-5p	581.19	264.69	-1.02
68	mmu-miR-221	427.73	854.05	1.00
69	mmu-miR-685	206.84	410.75	1.00
70	mmu-miR-25	18,186.61	9,035.65	-0.99
71	mmu-miR-1895	500.55	1,018.14	0.98
72	mmu-miR-200c	3,609.64	7,009.77	0.97
73	mmu-miR-26b	13,681.16	7,184.84	-0.95
74	mmu-miR-7a	636.87	325.87	-0.93
75	mmu-miR-222	1,187.77	2,134.70	0.93
76	mmu-miR-689	336.12	638.06	0.92
77	mmu-miR-183	224.06	440.34	0.92
78	mmu-miR-150*	202.72	382.23	0.92
79	mmu-miR-192	608.28	354.54	-0.82
80	mmu-miR-342-5p	276.89	453.48	0.79
81	mmu-miR-145	3,279.07	5,641.44	0.75
82	mmu-miR-451	514.31	273.80	-0.74
83	mmu-miR-21	12,663.09	20,552.40	0.73
84	mmu-miR-378	1,228.84	743.34	-0.72
85	mmu-miR-92b	11,917.59	7,661.74	-0.65
86	mmu-miR-30b	11,162.04	7,137.10	-0.63
87	mmu-miR-181a	27,025.20	17,595.62	-0.62
88	mmu-miR-203	1,269.50	1,910.78	0.60
89	mmu-miR-103	4,505.78	3,060.79	-0.52
90	mmu-miR-27b	6,093.96	4,130.32	-0.51
91	mmu-let-7b	20,738.60	30,063.24	0.51
92	mmu-miR-140*	1,862.50	1,312.60	-0.50
93	mmu-miR-126-3p	7,598.79	10,736.54	0.49
94	mmu-miR-23a	14,563.78	20,092.01	0.48
95	mmu-miR-361	3,953.08	2,856.35	-0.48
96	mmu-miR-16	26,788.15	19,302.94	-0.47

97	mmu-miR-720	847.11	1,176.98	0.47
98	mmu-miR-27a	3,291.63	2,391.22	-0.46
99	mmu-miR-107	3,856.69	2,891.00	-0.44
100	mmu-miR-23b	15,310.06	20,647.96	0.44
101	mmu-let-7c	23,780.39	32,850.76	0.44
102	mmu-let-7a	26,278.63	35,441.47	0.42
103	mmu-miR-320	2,452.77	3,065.99	0.40
104	mmu-miR-155	6,864.05	8,991.66	0.40
105	mmu-miR-30c	12,194.29	9,278.25	-0.38
106	mmu-miR-146a	5,334.25	6,827.01	0.36
107	mmu-miR-423-5p	3,292.57	2,506.28	-0.35
108	mmu-miR-24	6,281.25	7,929.42	0.34
109	mmu-miR-15b	28,207.30	22,643.68	-0.32
110	mmu-miR-29a	9,636.21	12,109.34	0.31
111	mmu-miR-30d	4,869.21	5,870.38	0.26
112	mmu-let-7g	21,574.10	19,053.34	-0.24
113	mmu-let-7d	24,268.24	28,506.86	0.22
114	mmu-let-7i	21,667.09	19,309.83	-0.20
115	mmu-let-7f	24,842.91	28,930.69	0.19
116	mmu-miR-26a	25,470.63	27,973.30	0.15

Chip: 03_M12.0_090444

Sample A: Thymus-LPS-3 – Cy3
Sample B: Thymus-Ctl-3 – Cy5

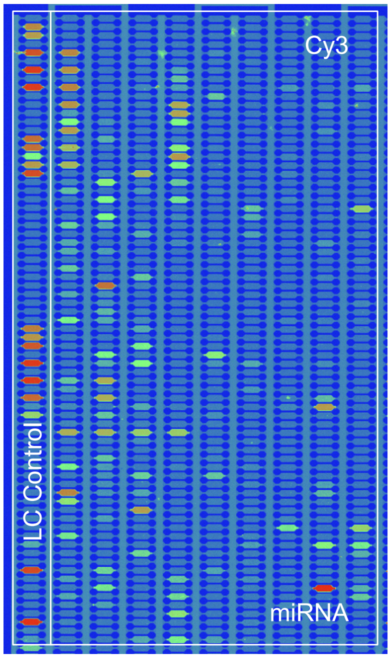

Sample A

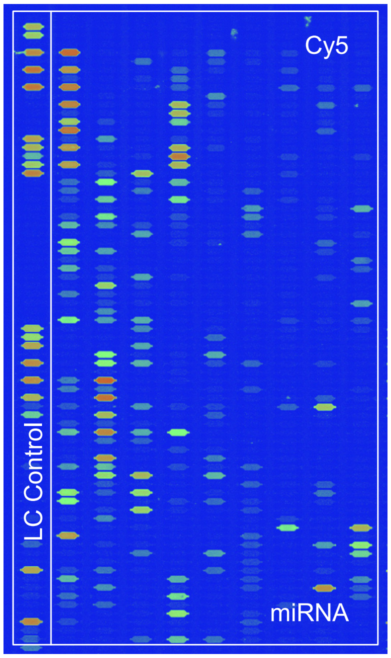

Sample B

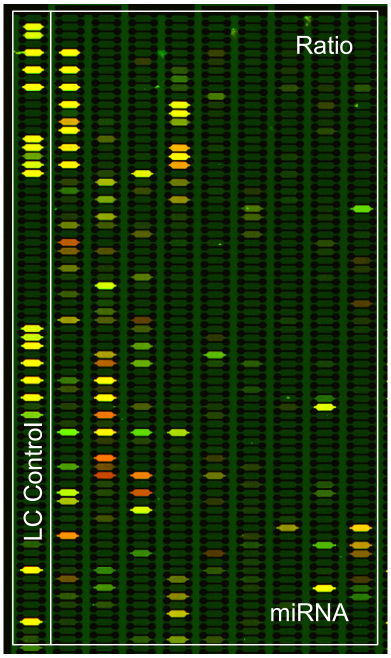

Cy3/Cy5

LC Sciences, LLC | www.LCsciences.com | support@LCsciences.com
2575 W. Bellfort, Suite 270, Houston, Texas 77054
Tel. 713 664-7087, Fax 713 664-8181

Table 3 Call list (differentially expressed transcripts with p-value < 0.01)

No.	Probe_ID	Sample A Signal	Sample B Signal	log2 (Sample B / Sample A)
1	mmu-miR-181d	377.98	9,487.68	4.56
2	mmu-miR-374	131.83	2,432.71	4.15
3	mmu-miR-20b	1,076.53	10,732.86	3.35
4	mmu-miR-340-5p	78.52	731.82	3.32
5	mmu-miR-30e*	37.40	372.13	3.30
6	mmu-miR-363	86.95	765.43	3.28
7	mmu-miR-467c	13.98	150.62	3.24
8	mmu-miR-15b*	108.02	1,019.05	3.10
9	mmu-miR-106a	1,087.32	9,165.91	3.08
10	mmu-miR-98	207.28	1,644.31	3.05
11	mmu-miR-301a	19.13	164.87	3.03
12	mmu-miR-19b	320.61	2,739.70	3.00
13	mmu-miR-805	275.19	2,090.00	2.85
14	mmu-miR-181b	2,779.90	19,872.24	2.83
15	mmu-miR-467e	46.44	258.95	2.80
16	mmu-miR-689	1,959.49	326.55	-2.65
17	mmu-miR-350	200.25	1,235.57	2.58
18	mmu-miR-99b	2,937.98	510.46	-2.54
19	mmu-miR-421	75.85	418.32	2.42
20	mmu-miR-15a	1,628.40	8,320.57	2.40
21	mmu-let-7d*	545.09	101.57	-2.37
22	mmu-miR-181a-1*	158.82	801.03	2.33
23	mmu-miR-17	2,951.86	13,884.19	2.30
24	mmu-miR-1224	15,320.29	3,032.66	-2.26
25	mmu-miR-20a	3,204.47	15,232.48	2.25
26	mmu-miR-181c	792.82	3,342.36	2.23
27	mmu-miR-205	11,860.64	2,521.95	-2.21
28	mmu-miR-1892	1,800.44	406.70	-2.19
29	mmu-miR-7a	233.64	1,126.13	2.17
30	mmu-miR-762	11,841.94	2,353.18	-2.16
31	mmu-miR-705	8,003.55	1,771.49	-2.14
32	mmu-miR-214	4,037.78	950.68	-2.09
33	mmu-miR-669a	56.34	255.59	2.06
34	mmu-miR-18a	198.17	868.88	2.05
35	mmu-miR-99a	1,581.89	387.67	-2.04
36	mmu-miR-100	2,326.04	530.72	-2.04
37	mmu-miR-148b	68.65	271.54	2.00
38	mmu-miR-128	5,282.02	20,499.63	1.93
39	mmu-miR-101b	76.85	264.75	1.92
40	mmu-miR-222	2,692.86	683.17	-1.91
41	mmu-miR-125a-5p	5,761.50	1,583.61	-1.86
42	mmu-miR-466j	87.84	265.48	1.81
43	mmu-miR-29b	69.73	247.39	1.81
44	mmu-miR-872	101.72	370.71	1.79
45	mmu-miR-342-3p	8,507.85	2,565.39	-1.75
46	mmu-miR-130b	934.68	3,390.05	1.74

47	mmu-miR-1894-3p	4,124.30	1,184.13	-1.74
48	mmu-miR-7a*	122.07	442.03	1.69
49	mmu-miR-93	1,643.74	5,181.12	1.62
50	mmu-miR-106b	1,327.57	4,060.77	1.57
51	mmu-miR-150	30,830.34	10,533.19	-1.52
52	mmu-miR-30e	303.43	900.22	1.47
53	mmu-miR-125b-5p	25,957.96	9,333.25	-1.46
54	mmu-miR-1195	2,204.60	812.24	-1.44
55	mmu-miR-451	1,218.80	466.40	-1.39
56	mmu-miR-322	77.74	203.11	1.39
57	mmu-miR-709	50,312.92	19,386.46	-1.38
58	mmu-miR-467a*	163.60	509.97	1.37
59	mmu-miR-151-3p	395.20	144.38	-1.36
60	mmu-miR-466f	196.75	553.47	1.36
61	mmu-miR-26b	6,603.90	16,573.41	1.34
62	mmu-miR-699	1,758.56	751.53	-1.32
63	mmu-miR-455	546.35	223.10	-1.27
64	mmu-miR-148a	743.19	1,735.19	1.26
65	mmu-miR-200b	5,324.24	2,216.13	-1.26
66	mmu-miR-466a-3p	225.93	543.56	1.26
67	mmu-miR-200c	7,070.76	2,906.02	-1.25
68	mmu-miR-698	707.27	335.62	-1.25
69	mmu-miR-466b-3-3p	217.76	515.97	1.21
70	mmu-miR-720	1,103.15	523.36	-1.19
71	mmu-miR-466f-3p	756.18	335.26	-1.15
72	mmu-miR-320	3,065.14	1,394.80	-1.11
73	mmu-miR-139-5p	387.24	168.28	-1.11
74	mmu-miR-532-5p	247.18	475.61	1.10
75	mmu-miR-150*	379.88	163.61	-1.07
76	mmu-miR-223	874.19	402.57	-1.02
77	mmu-miR-669c	388.33	750.94	0.95
78	mmu-miR-25	8,592.94	16,403.50	0.93
79	mmu-let-7e	6,562.14	11,772.78	0.92
80	mmu-miR-92b	5,506.62	10,488.38	0.91
81	mmu-miR-145	6,417.02	3,558.49	-0.91
82	mmu-miR-342-5p	452.70	242.07	-0.90
83	mmu-miR-21	19,790.09	11,437.99	-0.78
84	mmu-miR-423-5p	2,664.75	1,499.08	-0.76
85	mmu-miR-192	414.58	802.74	0.76
86	mmu-miR-132	1,136.73	673.80	-0.75
87	mmu-miR-22	896.66	536.15	-0.74
88	mmu-miR-221	903.11	495.50	-0.74
89	mmu-miR-690	21,539.11	12,664.76	-0.74
90	mmu-miR-151-5p	1,919.01	1,126.44	-0.64
91	mmu-miR-1895	829.23	538.00	-0.62
92	mmu-miR-24	6,862.67	4,471.41	-0.62
93	mmu-miR-425	1,532.00	999.91	-0.62
94	mmu-miR-15b	17,882.53	26,384.95	0.60
95	mmu-miR-195	1,439.35	2,069.12	0.58
96	mmu-miR-16	17,112.72	24,040.35	0.57

97	mmu-miR-29a	12,532.05	8,279.18	-0.56
98	mmu-miR-185	773.12	1,207.44	0.55
99	mmu-miR-92a	11,979.68	16,987.92	0.52
100	mmu-miR-181a	15,648.44	21,737.56	0.51
101	mmu-miR-199a-3p	2,898.28	2,172.60	-0.48
102	mmu-miR-23b	20,312.00	14,983.55	-0.47
103	mmu-miR-146a	6,559.84	4,710.88	-0.45
104	mmu-miR-23a	19,521.42	14,506.66	-0.44
105	mmu-miR-143	8,036.62	6,216.63	-0.41
106	mmu-miR-1187	4,579.37	6,089.18	0.41
107	mmu-let-7g	14,844.71	20,000.09	0.41
108	mmu-miR-30d	5,122.43	3,864.10	-0.39
109	mmu-miR-155	5,753.75	7,324.00	0.38
110	mmu-miR-191	16,154.01	13,375.29	-0.36
111	mmu-miR-574-5p	4,852.71	6,307.34	0.36
112	mmu-miR-361	2,765.03	3,597.80	0.35
113	mmu-miR-126-3p	9,542.58	7,329.23	-0.29
114	mmu-let-7i	16,273.95	19,671.67	0.29
115	mmu-let-7f	22,306.59	26,147.50	0.27
116	mmu-let-7a	26,287.74	28,770.82	0.13

Following is the probe layout of the above miRNA array images.

PUC2PM									
PUC2MM									
BKG0									
PUC2PM-20B	mmu-let-7a	mmu-miR-135a*	mmu-miR-1899	mmu-miR-22*	mmu-miR-30e	mmu-miR-378*	mmu-miR-467c	mmu-miR-666-3p	mmu-miR-715
PUC2MM-20B	mmu-let-7a*	mmu-miR-135b	mmu-miR-18a	mmu-miR-220	mmu-miR-30e*	mmu-miR-379	mmu-miR-467d	mmu-miR-666-5p	mmu-miR-717
a2-PUC2PM-20B	mmu-let-7b	mmu-miR-136	mmu-miR-18a*	mmu-miR-221	mmu-miR-31	mmu-miR-380-3p	mmu-miR-467e	mmu-miR-667	mmu-miR-718
a2-PUC2MM-20B	mmu-let-7b*	mmu-miR-136*	mmu-miR-18b	mmu-miR-222	mmu-miR-31*	mmu-miR-380-5p	mmu-miR-467e*	mmu-miR-668	mmu-miR-719
a3-PUC2PM-20B	mmu-let-7c	mmu-miR-137	mmu-miR-190	mmu-miR-223	mmu-miR-32	mmu-miR-381	mmu-miR-467f	mmu-miR-669a	mmu-miR-720
a3-PUC2MM-20B	mmu-let-7c-1*	mmu-miR-138	mmu-miR-1900	mmu-miR-224	mmu-miR-320	mmu-miR-382	mmu-miR-467g	mmu-miR-669b	mmu-miR-721
	mmu-let-7d	mmu-miR-138*	mmu-miR-1901	mmu-miR-23a	mmu-miR-322	mmu-miR-382*	mmu-miR-467h	mmu-miR-669c	mmu-miR-741
	mmu-let-7d*	mmu-miR-139-3p	mmu-miR-1902	mmu-miR-23b	mmu-miR-322*	mmu-miR-383	mmu-miR-468	mmu-miR-669d	mmu-miR-742
	mmu-let-7e	mmu-miR-139-5p	mmu-miR-1903	mmu-miR-24	mmu-miR-323-3p	mmu-miR-384-3p	mmu-miR-469	mmu-miR-669e	mmu-miR-742*
	mmu-let-7f	mmu-miR-140	mmu-miR-1904	mmu-miR-24-1*	mmu-miR-323-5p	mmu-miR-384-5p	mmu-miR-470	mmu-miR-669f	mmu-miR-743a
mmu-5S-b1	mmu-let-7f*	mmu-miR-140*	mmu-miR-1905	mmu-miR-24-2*	mmu-miR-324-3p	mmu-miR-409-3p	mmu-miR-470*	mmu-miR-669g	mmu-miR-743b-3p
mmu-5S-b2	mmu-let-7g	mmu-miR-141	mmu-miR-1906	mmu-miR-25	mmu-miR-324-5p	mmu-miR-409-5p	mmu-miR-471	mmu-miR-669h-3p	mmu-miR-743b-5p
mmu-5S-b3	mmu-let-7g*	mmu-miR-141*	mmu-miR-1907	mmu-miR-26a	mmu-miR-325	mmu-miR-410	mmu-miR-483	mmu-miR-669h-5p	mmu-miR-744
mmu-5S-b4	mmu-let-7i	mmu-miR-142-3p	mmu-miR-190b	mmu-miR-26b	mmu-miR-325*	mmu-miR-411	mmu-miR-483*	mmu-miR-669i	mmu-miR-744*
mmu-5S-b5	mmu-let-7i*	mmu-miR-142-5p	mmu-miR-191	mmu-miR-26b*	mmu-miR-326	mmu-miR-411*	mmu-miR-484	mmu-miR-669j	mmu-miR-758
	mmu-miR-1	mmu-miR-143	mmu-miR-191*	mmu-miR-27a	mmu-miR-327	mmu-miR-412	mmu-miR-485	mmu-miR-669k	mmu-miR-759
	mmu-miR-100	mmu-miR-144	mmu-miR-192	mmu-miR-27a*	mmu-miR-328	mmu-miR-421	mmu-miR-485*	mmu-miR-670	mmu-miR-760
	mmu-miR-101a	mmu-miR-145	mmu-miR-193	mmu-miR-27b	mmu-miR-329	mmu-miR-423-3p	mmu-miR-486	mmu-miR-671-3p	mmu-miR-761
	mmu-miR-101a*	mmu-miR-145*	mmu-miR-193*	mmu-miR-27b*	mmu-miR-33	mmu-miR-423-5p	mmu-miR-487b	mmu-miR-671-5p	mmu-miR-762
	mmu-miR-101b	mmu-miR-146a	mmu-miR-193b	mmu-miR-28	mmu-miR-33*	mmu-miR-425	mmu-miR-488	mmu-miR-672	mmu-miR-763
	mmu-miR-103	mmu-miR-146b	mmu-miR-194	mmu-miR-28*	mmu-miR-330	mmu-miR-425*	mmu-miR-488*	mmu-miR-673-3p	mmu-miR-764-3p
	mmu-miR-105	mmu-miR-146b*	mmu-miR-195	mmu-miR-290-3p	mmu-miR-330*	mmu-miR-429	mmu-miR-489	mmu-miR-673-5p	mmu-miR-764-5p
	mmu-miR-106a	mmu-miR-147	mmu-miR-196a	mmu-miR-290-5p	mmu-miR-331-3p	mmu-miR-431	mmu-miR-490	mmu-miR-674	mmu-miR-770-3p
	mmu-miR-106b	mmu-miR-148a	mmu-miR-196a*	mmu-miR-291a-3p	mmu-miR-331-5p	mmu-miR-431*	mmu-miR-491	mmu-miR-674*	mmu-miR-770-5p
	mmu-miR-106b*	mmu-miR-148a*	mmu-miR-196b	mmu-miR-291a-5p	mmu-miR-335-3p	mmu-miR-433	mmu-miR-493	mmu-miR-675-3p	mmu-miR-7a
	mmu-miR-107	mmu-miR-148b	mmu-miR-197	mmu-miR-291b-3p	mmu-miR-335-5p	mmu-miR-433*	mmu-miR-494	mmu-miR-675-5p	mmu-miR-7a*
	mmu-miR-10a	mmu-miR-149	mmu-miR-199a-3p	mmu-miR-291b-5p	mmu-miR-337-3p	mmu-miR-434-3p	mmu-miR-495	mmu-miR-676	mmu-miR-7b
	mmu-miR-10a*	mmu-miR-150	mmu-miR-199a-5p	mmu-miR-292-3p	mmu-miR-337-5p	mmu-miR-434-5p	mmu-miR-496	mmu-miR-676*	mmu-miR-802
	mmu-miR-10b	mmu-miR-150*	mmu-miR-199b*	mmu-miR-292-5p	mmu-miR-338-3p	mmu-miR-448	mmu-miR-497	mmu-miR-677	mmu-miR-804
	mmu-miR-10b*	mmu-miR-151-3p	mmu-miR-19a	mmu-miR-293	mmu-miR-338-5p	mmu-miR-449a	mmu-miR-499	mmu-miR-678	mmu-miR-805
	mmu-miR-1186	mmu-miR-151-5p	mmu-miR-19a*	mmu-miR-293*	mmu-miR-339-3p	mmu-miR-449b	mmu-miR-500	mmu-miR-679	mmu-miR-871
BKG0	mmu-miR-1187	mmu-miR-152	mmu-miR-19b	mmu-miR-294	mmu-miR-339-5p	mmu-miR-449c	mmu-miR-501-3p	mmu-miR-680	mmu-miR-872
PUC2PM	mmu-miR-1188	mmu-miR-153	mmu-miR-200a	mmu-miR-294*	mmu-miR-340-3p	mmu-miR-450a-3p	mmu-miR-501-5p	mmu-miR-681	mmu-miR-872*
PUC2MM	mmu-miR-1190	mmu-miR-154	mmu-miR-200a*	mmu-miR-295	mmu-miR-340-5p	mmu-miR-450a-5p	mmu-miR-503	mmu-miR-682	mmu-miR-873
PUC2PM-20B	mmu-miR-1191	mmu-miR-154*	mmu-miR-200b	mmu-miR-295*	mmu-miR-341	mmu-miR-450b-3p	mmu-miR-503*	mmu-miR-683	mmu-miR-874
PUC2MM-20B	mmu-miR-1192	mmu-miR-155	mmu-miR-200b*	mmu-miR-296-3p	mmu-miR-342-3p	mmu-miR-450b-5p	mmu-miR-504	mmu-miR-684	mmu-miR-875-3p
a2-PUC2PM-20B	mmu-miR-1193	mmu-miR-15a	mmu-miR-200c	mmu-miR-296-5p	mmu-miR-342-5p	mmu-miR-451	mmu-miR-505	mmu-miR-685	mmu-miR-875-5p
a2-PUC2MM-20B	mmu-miR-1194	mmu-miR-15a*	mmu-miR-200c*	mmu-miR-297a	mmu-miR-343	mmu-miR-452	mmu-miR-509-3p	mmu-miR-686	mmu-miR-876-3p
a3-PUC2PM-20B	mmu-miR-1195	mmu-miR-15b	mmu-miR-201	mmu-miR-297a*	mmu-miR-344	mmu-miR-453	mmu-miR-509-5p	mmu-miR-687	mmu-miR-876-5p
a3-PUC2MM-20B	mmu-miR-1196	mmu-miR-15b*	mmu-miR-202-3p	mmu-miR-297b-5p	mmu-miR-345-3p	mmu-miR-455	mmu-miR-511	mmu-miR-688	mmu-miR-877
a-PUC2PM	mmu-miR-1197	mmu-miR-16	mmu-miR-202-5p	mmu-miR-297c	mmu-miR-345-5p	mmu-miR-455*	mmu-miR-532-3p	mmu-miR-689	mmu-miR-877*
a-PUC2MM2d	mmu-miR-1198	mmu-miR-16*	mmu-miR-203	mmu-miR-298	mmu-miR-346	mmu-miR-463	mmu-miR-532-5p	mmu-miR-690	mmu-miR-878-3p
Ctr01-3P	mmu-miR-1199	mmu-miR-17	mmu-miR-203*	mmu-miR-299	mmu-miR-34a	mmu-miR-463*	mmu-miR-539	mmu-miR-691	mmu-miR-878-5p
Ctr01-3M05	mmu-miR-122	mmu-miR-17*	mmu-miR-204	mmu-miR-299*	mmu-miR-34b-3p	mmu-miR-464	mmu-miR-540-3p	mmu-miR-692	mmu-miR-879
Ctr01-3M10	mmu-miR-1224	mmu-miR-181a	mmu-miR-205	mmu-miR-29a	mmu-miR-34b-5p	mmu-miR-465a-3p	mmu-miR-540-5p	mmu-miR-693-3p	mmu-miR-879*
Ctr02-3P	mmu-miR-124	mmu-miR-181a-1*	mmu-miR-206	mmu-miR-29a*	mmu-miR-34c	mmu-miR-465a-5p	mmu-miR-541	mmu-miR-693-5p	mmu-miR-880
Ctr02-3M05	mmu-miR-124*	mmu-miR-181a-2*	mmu-miR-207	mmu-miR-29b	mmu-miR-34c*	mmu-miR-465b-5p	mmu-miR-542-3p	mmu-miR-694	mmu-miR-881
Ctr02-3M10	mmu-miR-125a-3p	mmu-miR-181b	mmu-miR-208a	mmu-miR-29b*	mmu-miR-350	mmu-miR-465c-5p	mmu-miR-542-5p	mmu-miR-695	mmu-miR-881*
Ctr03-3P	mmu-miR-125a-5p	mmu-miR-181c	mmu-miR-208b	mmu-miR-29c	mmu-miR-351	mmu-miR-466a-3p	mmu-miR-543	mmu-miR-696	mmu-miR-882
Ctr03-3M05	mmu-miR-125b*	mmu-miR-181d	mmu-miR-20a	mmu-miR-29c*	mmu-miR-361	mmu-miR-466a-5p	mmu-miR-544	mmu-miR-697	mmu-miR-883a-3p
Ctr03-3M10	mmu-miR-125b-3p	mmu-miR-182	mmu-miR-20a*	mmu-miR-300	mmu-miR-362-3p	mmu-miR-466b-3-3p	mmu-miR-546	mmu-miR-698	mmu-miR-883a-5p
Ctr04-3P	mmu-miR-125b-5p	mmu-miR-183	mmu-miR-20b	mmu-miR-300*	mmu-miR-362-5p	mmu-miR-466b-5p	mmu-miR-547	mmu-miR-699	mmu-miR-883b-3p
Ctr04-3M05	mmu-miR-126-3p	mmu-miR-183*	mmu-miR-20b*	mmu-miR-301a	mmu-miR-363	mmu-miR-466c-5p	mmu-miR-551b	mmu-miR-700	mmu-miR-883b-5p
Ctr04-3M10	mmu-miR-126-5p	mmu-miR-184	mmu-miR-21	mmu-miR-301b	mmu-miR-365	mmu-miR-466d-3p	mmu-miR-568	mmu-miR-701	mmu-miR-9
Ctr05-3P	mmu-miR-127	mmu-miR-185	mmu-miR-21*	mmu-miR-302a	mmu-miR-367	mmu-miR-466d-5p	mmu-miR-574-3p	mmu-miR-702	mmu-miR-9*
Ctr05-3M05	mmu-miR-127*	mmu-miR-186	mmu-miR-210	mmu-miR-302a*	mmu-miR-369-3p	mmu-miR-466e-5p	mmu-miR-574-5p	mmu-miR-703	mmu-miR-92a
Ctr05-3M10	mmu-miR-128	mmu-miR-186*	mmu-miR-211	mmu-miR-302b	mmu-miR-369-5p	mmu-miR-466f	mmu-miR-582-3p	mmu-miR-704	mmu-miR-92a*
Ctr06-3P	mmu-miR-129-3p	mmu-miR-187	mmu-miR-212	mmu-miR-302b*	mmu-miR-370	mmu-miR-466f-3p	mmu-miR-582-5p	mmu-miR-705	mmu-miR-92b
Ctr06-3M05	mmu-miR-129-5p	mmu-miR-188-3p	mmu-miR-214	mmu-miR-302c	mmu-miR-374	mmu-miR-466f-5p	mmu-miR-590-3p	mmu-miR-706	mmu-miR-93
Ctr06-3M10	mmu-miR-1-2-as	mmu-miR-188-5p	mmu-miR-214*	mmu-miR-302c*	mmu-miR-374*	mmu-miR-466g	mmu-miR-590-5p	mmu-miR-707	mmu-miR-93*
Ctr07-3P	mmu-miR-130a	mmu-miR-1892	mmu-miR-215	mmu-miR-302d	mmu-miR-375	mmu-miR-466h	mmu-miR-592	mmu-miR-708	mmu-miR-96
Ctr07-3M05	mmu-miR-130b	mmu-miR-1893	mmu-miR-216a	mmu-miR-30a	mmu-miR-376a	mmu-miR-466i	mmu-miR-598	mmu-miR-708*	mmu-miR-98
Ctr07-3M10	mmu-miR-130b*	mmu-miR-1894-3p	mmu-miR-216b	mmu-miR-30a*	mmu-miR-376a*	mmu-miR-466j	mmu-miR-615-3p	mmu-miR-709	mmu-miR-99a
Ctr08-3P	mmu-miR-132	mmu-miR-1894-5p	mmu-miR-217	mmu-miR-30b	mmu-miR-376b	mmu-miR-466k	mmu-miR-615-5p	mmu-miR-710	mmu-miR-99b
Ctr08-3M05	mmu-miR-133a	mmu-miR-1895	mmu-miR-218	mmu-miR-30b*	mmu-miR-376b*	mmu-miR-466l	mmu-miR-652	mmu-miR-711	mmu-miR-99b*
Ctr08-3M10	mmu-miR-133a*	mmu-miR-1896	mmu-miR-218-1*	mmu-miR-30c	mmu-miR-376c	mmu-miR-467a	mmu-miR-653	mmu-miR-712	
Ctr09-3P	mmu-miR-133b	mmu-miR-1897-3p	mmu-miR-218-2*	mmu-miR-30c-1*	mmu-miR-376c*	mmu-miR-467a*	mmu-miR-654-3p	mmu-miR-712*	
Ctr09-3M05	mmu-miR-134	mmu-miR-1897-5p	mmu-miR-219	mmu-miR-30c-2*	mmu-miR-377	mmu-miR-467b	mmu-miR-654-5p	mmu-miR-713	
Ctr09-3M10	mmu-miR-135a	mmu-miR-1898	mmu-miR-22	mmu-miR-30d	mmu-miR-378	mmu-miR-467b*	mmu-miR-665	mmu-miR-714	

IV.Data Analysis

We provide the result of a data analysis in data files (Chip#_Data.xls). There are seven worksheets in each file as described in the following.

- Worksheet "File Info" – provides information on data files, samples, and data analysis parameters.
- Worksheet "Simple Differential" – lists all differentially expressed transcripts with p-value < 0.01. Mature miRNAs are sorted separately according to differential ratios. The ratio values are presented in log_2 scale for quick and easy assessing differential direction as well as magnitude. A positive log_2 value indicates an upper regulation and a negative log_2 value indicates a down regulation. One can easily convert a log_2 value into a arithmetic ratio on a calculator by typing in 2^(value). Detailed data processing statistics are listed in Worksheet "Differential Data".
- Worksheet "Simple Detectable" – lists average signal values all transcripts on the array. The signal values are derived by background subtraction and normalization. Blank spaces represent signal values below detection level. A transcript to be listed as detectable must meets at least two conditions: signal intensity higher than 3×(background standard deviation) and spot CV < 0.5. CV is calculated by (standard deviation)/(signal intensity). When repeating probes are present on an array, a transcript is listed as detectable only if the signals from at least 50% of the repeating probes are above detection level. Detailed data processing statistics are listed in Worksheet "Detectable Transcripts".
- Worksheet "Raw Data" – lists raw data extracted from image files with corresponding probe, sequence, and location information.
- Worksheet "Processed Data" – lists processed data, including background-subtracted and normalized signals, p-values, statistically significant log ratios of Cy3 and Cy5 labeled transcripts, and a scatter plot of the processed data.
- Worksheet "Differential Data" – lists all differentially expressed transcripts with p-value < 0.01 along with data processing statistics. Signals are listed in median signal values of repeating probes of p-value < 0.01 that are listed in the upper portion of the table on Worksheet "Processed Data". Median values are used to minimize the effect of occasional "non-uniform spots" that may have signal values deviate from average signal values but have p-values < 0.01.
- Worksheet "Detectable Transcripts" – lists all the transcripts with signals above detection levels along with data processing statistics. Signal intensities are listed in average values of repeating spots. During data process, "bad spots" that have signal values deviated more than 50% of average values of repeating spots and/or spot CV larger than 0.5 are discarded.

In data file package a probe layout file containing a complete list of probe positions and target sequences is included in the file directory of each chip.

V. Suggestions for Data Analysis

In case you want to perform your own data analysis we have the following suggestions.

1. Background should be calculated from the median of 5% to 25% of low intensity cells. BKG0 and blank cells should be excluded for the background calculation.
2. All "Production_Use_Probes" (including BKG0, PUC2 …,) and blank cells which are listed in a supplied layout file, should be excluded during data normalization.
3. The systematic dye bias has been found on the following probes. Therefore, it is advised to exclude the data of these probes from further consideration.

   Probes
   - mmu-miR-341
   - mmu-miR-377
